# Thermochemical Co-Valorization of Sewage Sludge and Plastic Waste: Co-Processing and Sludge-Derived Catalysis

**DOI:** 10.3390/polym18202548

**Published:** 2026-10-16

**Authors:** Nazar Zabara, Diyar Tokmurzin, Aigerim Kaiaidarova, Fedor Pogorov, Akezhan Balgarayev, Valeriy Panyukov, Yelena Vitoshnova, Sergey Efremov, Sergey Nechipurenko

**Affiliations:** 1 Center of Physical-Chemical Methods of Research and Analysis, Farabi University, Almaty 050012, Kazakhstan; nzr.zabara@gmail.com (N.Z.); aigerim_ko@list.ru (A.K.); fpogorov@gmail.com (F.P.); akezhanbalgarayev@gmail.com (A.B.); valeriypan0408@gmail.com (V.P.); helenvit4@gmail.com (Y.V.); efremsa@mail.ru (S.E.); nechipurenkos@mail.ru (S.N.); 2 Laboratory for Chemical Technology, Industriele Scheikunde, Ghent University, Technologiepark-Zwijnaarde 125, 9052 Gent, Belgium

**Keywords:** plastic waste, sewage sludge, co-pyrolysis, co-gasification, hydrothermal carbonization, hydrothermal liquefaction, sewage sludge char, sewage sludge ash, waste-derived catalysts, thermochemical conversion

## Abstract

Plastic waste and sewage sludge (SS) are abundant, heterogeneous waste streams whose utilization and recycling are constrained by contamination, variable composition, and limited resource recovery. This review evaluates their integrated thermochemical conversion, focusing on co-incineration, co-pyrolysis, co-gasification, hydrothermal co-carbonization, hydrothermal co-liquefaction, and the use of SS-derived char (SSC) and sewage sludge ash (SSA) as catalytic materials. Co-processing benefits arise from complementary feedstock properties: plastics provide high carbon, hydrogen, volatile matter (VM), and heating value, whereas SS contributes oxygenated intermediates and mineral phases containing Si, Al, Fe, Ca, Mg, and P. Among the reviewed routes, co-pyrolysis shows the strongest evidence of non-additive interactions, including hydrogen transfer, altered decomposition kinetics, and changes in liquid composition. Co-gasification can enhance H_2_ formation and tar conversion, while hydrothermal routes are especially attractive for wet SS but remain comparatively underexplored. The review further distinguishes SSA, dominated by mineral acid–base and redox functionality, from SSC, which combines these mineral effects with adsorption and surface reactions on a porous carbon matrix. Key barriers include catalyst deactivation, chlorine and sulfur poisoning, heavy-metal migration, feedstock variability, and limited pilot-scale validation. Future development requires mechanistic controls, catalyst stability testing, techno-economic analysis, life-cycle assessment, and integrated nutrient recovery to establish practical circular waste-management systems.

## 1. Introduction

**Figure 1:**
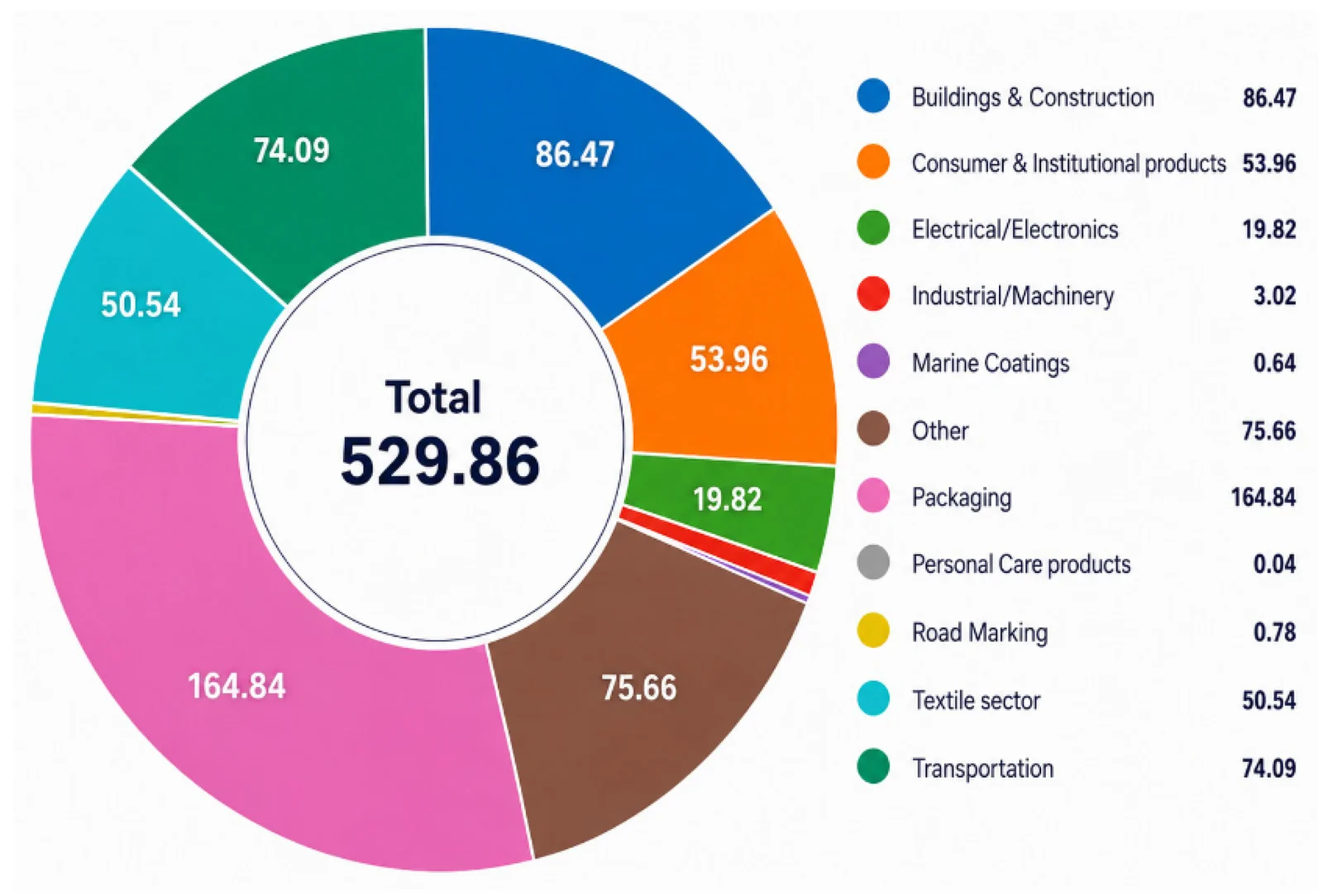
Global plastic-use destinations worldwide based on OECD projection for year 2026 [5].

Plastics have become indispensable because they combine low density, durability, chemical and moisture resistance, and versatility [1,2]. In 2023, global plastic production reached approximately 359 million tons annually, while plastic waste generation reached 389.33 million tons [1,3]. In terms of polymer types, plastic production is generally distributed as: polyethylene (PE) (26%), polypropylene (PP) (19%), polyvinyl chloride (PVC) (13%), polyethylene terephthalate (PET) (2%), polyurethane (PUR) (5%), and polystyrene (PS) (5%) [4]. As shown in Figure 1, packaging is the largest plastic-consuming sector and includes many short-lived products [5].

A substantial proportion of plastic waste remains outside material recovery loops [4,6]. Its persistence contributes to micro- and nanoplastic formation, while additives and degradation products may contaminate soil and groundwater [7,8].

Mechanical and chemical recycling recover valuable materials (Table 1), whereas incineration primarily reduces waste volume and recovers energy. Inadequate combustion control or flue-gas treatment can result in hazardous emissions [9,10]. Chemical recycling converts polymer chains into smaller molecules, fuels, monomers, or chemical feedstocks [11]. Pyrolysis and gasification can accommodate heterogeneous and contaminated wastes, creating opportunities for co-processing with sewage sludge (SS), although their energy requirements remain substantial.

Sewage sludge can be utilized through agricultural application and composting to recycle nutrients and organic matter, anaerobic digestion to recover biogas, and incineration to recover energy and reduce waste volume. Pyrolysis and gasification provide additional routes to gaseous products and carbonaceous or mineral residues, while hydrothermal processing enables the conversion of wet sludge. These technologies recover different components of the sludge, and their suitability depends on its composition, contaminant content, energy requirements, and opportunities for using the resulting products [18,19].

Producing catalytic materials represents a complementary option for valorizing sludge-derived solids rather than a preferred destination for all sewage sludge. Char and ash generated during thermal treatment may retain carbon surfaces and mineral constituents that can be used in subsequent conversion processes. Their functional value is demonstrated by the use of sludge-derived char to promote aromatic formation during waste-plastic pyrolysis [20]. This creates an opportunity to link sludge treatment with plastic valorization, potentially obtaining additional value from the solid residue. However, this application must be assessed against alternative residue uses, including phosphorus recovery, and must account for catalyst preparation, activity, lifetime, contaminant release, and final disposal. The relevant question is therefore under which conditions sludge-derived catalysis provides an additional benefit within an integrated treatment system [19].

SS–plastic co-valorization may improve feedstock availability, energy recovery, and product quality through complementary feedstock properties and interactions between decomposition intermediates. SS contains carbonaceous matter and mineral phases that may provide acidic, basic, and metallic sites capable of promoting cracking, reforming, deoxygenation, aromatization, and tar conversion [20,21]. However, changes in conversion kinetics or product distribution should not automatically be attributed to chemical synergy between the feedstocks, because they may also result from the catalytic activity of sludge minerals, altered heat and mass transfer, or changes in vapor residence time. Distinguishing between these effects is therefore essential for evaluating the actual benefits of SS–plastic co-processing.

Insufficient waste-plastic stream volumes can make plastic valorization plants uneconomic [22,23]. To improve economics of scale, co-feeding with biomass including agricultural wastes was proposed, but agricultural waste use incurs additional logistic expenses. SS streams have large volumes, already concentrated in water treatment facilities, and a relatively homogeneous composition. However, none of the previous studies considered the effect of co-feeding SS on techno-economic analysis (TEA) of plastic pyrolysis facilities.

Previous reviews addressed thermochemical plastic recycling [24,25] or sewage sludge valorization [26,27] and catalytic conversion separately [28,29]. However, no previous study specifically addressed the co-valorization of plastics and SS, and the use of SS-derived solid residual materials for plastic valorization. The specific research gap addressed in this review is the lack of an integrated framework that critically distinguishes genuine SS–plastic feedstock interactions from mineral-catalyzed and transport-related effects while simultaneously considering sewage sludge as both a co-feedstock and a precursor of catalytic materials. This review examines the direct thermochemical co-valorization of sewage sludge and plastics, as well as the conversion of sewage sludge into catalytic materials for upgrading plastic-derived products in an integrated manner. Its distinctive contribution is the simultaneous consideration of sewage sludge as both a co-feedstock and a source of catalytic materials, together with a critical distinction between genuine non-additive feedstock interactions, mineral-catalyzed effects, and transport-related effects. Particular emphasis is placed on co-pyrolysis and co-gasification and on the use of SS-derived char, ash, activated residues, and metal-modified catalysts. Their effects on cracking, reforming, aromatization, deoxygenation, tar reduction, and syngas production are critically assessed, together with catalyst deactivation, contaminant migration, and practical limitations. Overall, the review evaluates the potential of integrating plastic waste and SS valorization within a circular waste-management framework.

## 2. Types of Waste-Plastic Feedstock for Thermochemical Conversion

**Figure 2:**
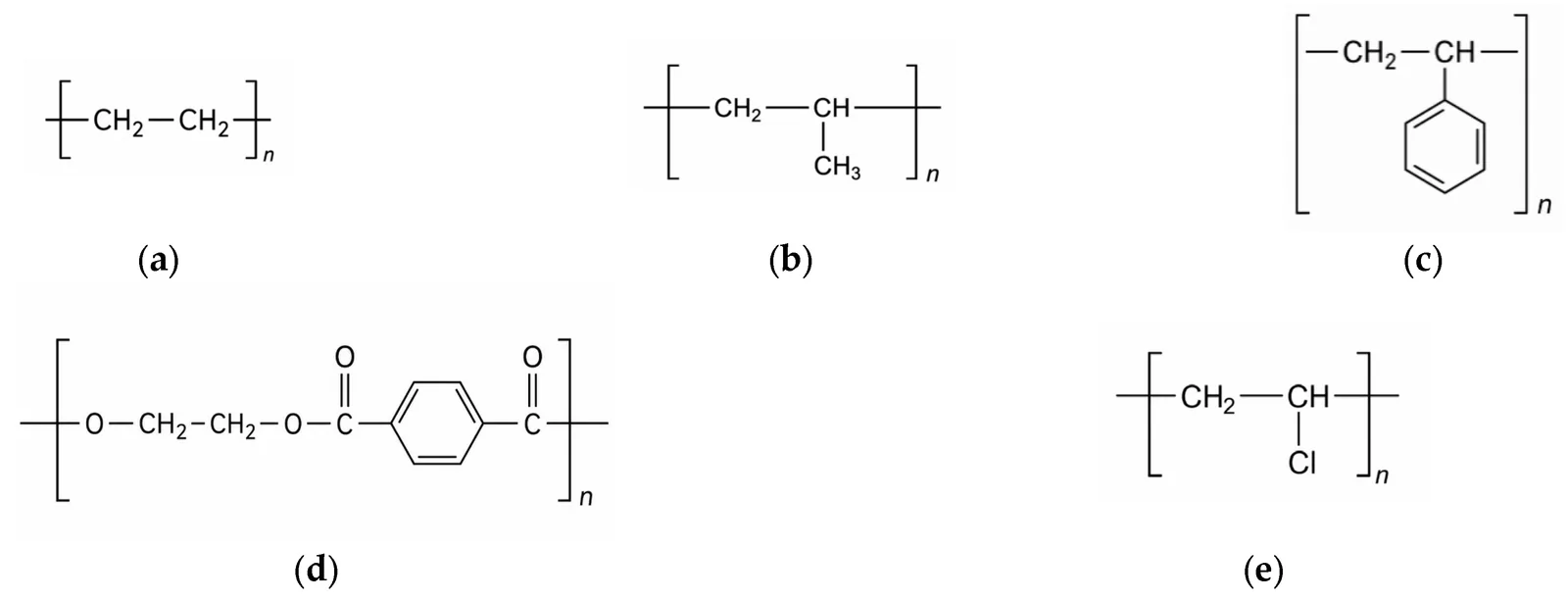
Structural formulas of (**a**) PE, (**b**) PP, (**c**) PS, (**d**) PET, and (**e**) PVC.

Feedstock characteristics strongly influence the yield distribution and composition of thermochemical conversion products. Plastics’ characteristics are mainly expressed through their elemental composition, moisture, VM, ash and fixed-carbon contents, polymer structure, and the presence of heteroatoms such as O, N, S, and Cl. Generally, plastics are characterized by high volatile-matter content and low ash and fixed-carbon contents (See Table 2). Their conversion behavior is substantially affected by polymer structure, illustrated in Figure 2. The major plastics occurring in municipal solid waste are PE (including HDPE and LDPE), PP, PS, PET and PVC. These polymers have consequently received the most attention in chemical recycling and valorization studies. Polyolefins, particularly PE and PP, are among the most favorable feedstocks due to their composition comprising mainly carbon and hydrogen, as well as the absence of solid residue including fixed carbon (FC) and ash. Due to its structure, PS thermolysis generates particularly high liquid yields dominated by styrene, together with ethylbenzene, toluene, and other aromatic hydrocarbons. PET thermolysis, considering its oxygenated structure, generates oxygenated aromatic compounds, including substantial quantities of benzoic-acid-related species, as well as CO and CO_2_. These oxygenates increase acidity and reduce the suitability of untreated pyrolysis liquids as fuels. PVC undergoes dechlorination between 200 and 320 °C, followed by decomposition and aromatization of the remaining carbonaceous structure. Then, Cl reacts with moisture and produces HCl, causing corrosion and the contamination of pyrolysis oils and gases. Therefore, PVC requires dechlorination, staged pyrolysis, alkaline sorbents, or downstream gas cleaning. Overall, PE, PP, and PS are generally the most favorable plastics for pyrolysis and thermochemical recycling, whereas PET offers opportunities for both depolymerization and thermochemical conversion, and PVC requires dedicated chlorine management.

## 3. SS Residue

**Figure 3:**
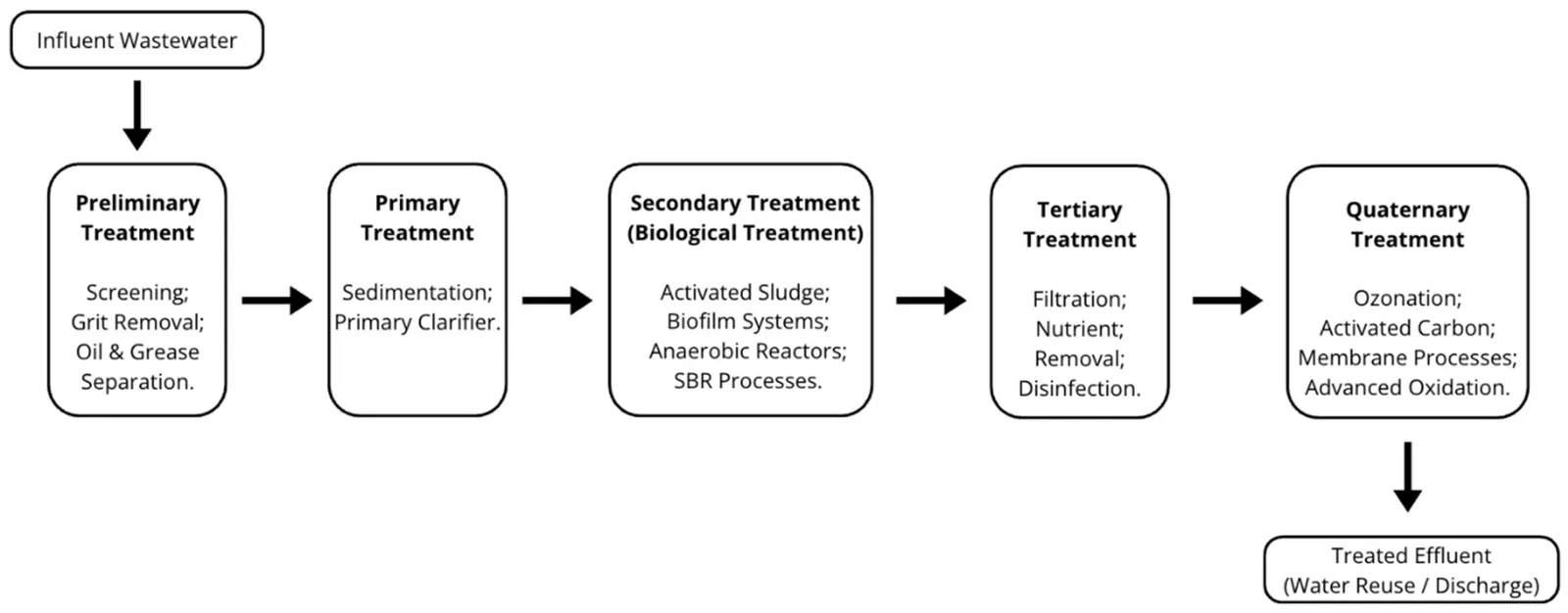
Stages of wastewater treatment.

Wastewater treatment typically combines mechanical, biological, and physicochemical processes arranged into preliminary, primary, secondary, tertiary, and, when required, quaternary stages, as illustrated in Figure 3 [19,33,34,35,36]. The number, sequence, and intensity of these stages depend on wastewater composition, treatment objectives, regulatory requirements, and economic feasibility. These processes generate SS, a semi-solid by-product of municipal and industrial wastewater treatment that generally requires dewatering or drying before disposal or further utilization [37,38]. Depending on its origin, SS may contain toxic metals, pesticides, fertilizers, food residues, per- and polyfluoroalkyl substances (PFAS), volatile organic compounds, polycyclic aromatic hydrocarbons, polychlorinated biphenyls, phthalates, and other hazardous contaminants [33,38,39].

### 3.1. SS Composition

SS composition is substantially affected by the origin of the wastewater, the types of discharges (household, commercial, and industrial), and pretreatment (drying, digestion). Sludge generated at different treatment stages may therefore differ markedly in both chemical and mineralogical composition as summarized in Table 3, showing proximate and ultimate analyses of SS.

Table 4 compares sewage sludge ash (SSA) obtained after SS devolatilization and incineration, including VM and FC. SSA is generally characterized by a complex mineral composition dominated by SiO_2_, Al_2_O_3_, Fe_2_O_3_, CaO, and P_2_O_5_. Minor components may include MgO, K_2_O, Na_2_O, SO_3_, ZnO, TiO_2_, CuO, and Cl-containing species [40]. The content and chemical form of these constituents depend strongly on the origin of the SS and the conversion conditions. These naturally accumulated metals may provide catalytically active sites or serve as precursors to active mineral phases in SS-derived catalysts. However, their actual catalytic contribution depends on their chemical form, dispersion, accessibility, and transformation during thermal treatment. Consequently, SS-derived materials have been investigated as catalysts or catalyst precursors in several applications, including catalytic cracking [41], electrocatalysis [42,43], hydrotreatment [44] and pyrolysis [45]. However, their catalytic performance depends not only on bulk composition but also on mineral phase, surface area, porosity, metal speciation, and accessibility of the active sites. Table 5 summarizes the metal concentrations in SS, which differ substantially among regions depending on local industrial activities [19,35].

**Table 3:** Typical proximate analysis of different SS types, compiled from the literature.

Study	SS Condition/Type	W, (wt%)	VM, (wt%, db)	A, (wt%, db)	FC, (wt%, db)	C (wt%, db)	H (wt%, db)	N (wt%, db)	S (wt%, db)	O (wt%, db)
Praspaliauskas and Pedišius (2017) [46]	Dried, dewatered and anaerobically digested/raw SS	2.23–88.47	47.00–67.30	21.60–41.02	11.10–18.71	34.31–46.56	4.49–6.52	4.35–7.24	0.99–1.83	11.65–19.53
Chan and Wang (2016) [47]	Primary, secondary, raw, dewatered and dried SS	4.65–98.40	47.56–71.07	13.77–48.01	4.43–16.24	27.44–43.98	4.07–6.56	3.21–7.55	4.26–5.62	20.85–30.87
Messaoudi et al. (2024) [48]	Dried SS	2.44	48.54	37.96–37.98	11.04–11.06	33.55	4.44–4.49	3.86–3.93	1.36–1.39	18.74–18.75
Dupont et al. (2025) [49]	Thermally dried SS, 12 monthly samples	1.35–30.61	35.39–52.47	30.62–45.68	2.28–6.59	23.40–29.80	3.95–6.03	3.34–4.14	2.77–5.38	25.26–28.32
Rijo et al. (2024), literature compilation [50]	Various SS types	71–89	22–82	11–76	1–22	26–69	4–9	2–9	0.2–2	22–56

**Table 4:** Comparison of SS inorganic fraction compositions.

Material	Ash Fraction of SSC [51]	Incinerated SSA [52]	SSA [53]	SS–Limestone Incineration Ashes [54]
SiO_2_ (wt%)	31.5	31.15	29.9	16.7–22.0
Al_2_O_3_ (wt%)	15.8	11.88	9.6	2.5–7.7
Fe_2_O_3_ (wt%)	10.4	23.95	22.5	4.9–15.4
CaO (wt%)	25.1	9.73	13.5	30.8–47.8
P_2_O_5_ (wt%)	—	9.27	18	10.3–12.7
MgO (wt%)	4.1	1.66	—	1.5–1.9
K_2_O (wt%)	0.9	3.53	—	1.3–1.6
Na_2_O (wt%)	1	2.42	1.0	—
SO_3_ (wt%)	—	4.08	0.1	4.7–5.6
Other reported components	SSC ash content: 89.3wt%	ZnO 0.89; TiO_2_ 0.61; CuO 0.24	Cl 0.15	Four different SSA samples

**Table 5:** Metals in SS [55,56,57,58,59].

Sample	Total Al (wt%)	Total Ca (wt%)	Total Fe (wt%)	Total K (wt%)	Total Na (wt%)	Total Mg (wt%)	Total Si (wt%)
Dry SS	2.1	5.1	3.0	0.5	0.1	0.6	12.7
	3.3	0.89	0.38	0.16	0.05	0.09	1.7
Wet SS	1.7–9.0	0.8–10	0.5–5.1	0.15–0.3	0.1–0.2	0.1–1.40	1.5–3.5
**Sample**	**Total Cd (mg/kg)**	**Total Cr (mg/kg)**	**Total Cu (mg/kg)**	**Total Ni (mg/kg)**	**Total Zn (mg/kg)**	**Total Pb (mg/kg)**	**Total Hg (mg/kg)**
Dry SS	0.2–61	24–1400	110–7400	7–540	170–28400	6–332	22
Wet SS	0.8–5.2	17.4–282	126–1720	27–82	281–1530	35–226	10

P, Al, Ca, Fe, and Si are among the major elements present in wet and dry SS. The reported P content of approximately 5–9% highlights its potential agronomic and resource-recovery value [60]. The inorganic fraction may contain phosphate-bearing crystalline phases such as CaK_2_P_2_O_7_, MgKPO_4_, and CaKPO_4_ [55,57]. Haider et al. (2022) identified 380 volatile organic compounds (VOCs) in SS using high-resolution PTR-Qi-ToF-MS [61]. Raw undigested SS (UDSS) emitted 233 VOCs, including 93 compounds unique to this material, whereas anaerobically digested SS (DSS) contained 193 VOCs. Subsequent drying to 30% and 60% dry-matter content resulted in 192 and 186 detected VOCs, respectively. UDSS also emitted aromatic compounds and indoles, including skatole, some of which may contribute to atmospheric aerosol formation [61]. Separately, the volatile-solids fraction of raw SS has been reported to vary widely, from approximately 14% to 83% of total solids, depending on its origin and treatment history.

Thickening, dewatering, and thermal treatment progressively concentrate the inorganic fraction of SS. Mechanical dewatering can increase ash content to 32.4 ± 11.8% of total solids compared with 3.3 ± 1.7% in thickened sludge [60]. After thermochemical treatment, the solid residue may contain approximately 75–100 wt% ash [59]. Pyrolysis produces SSC containing residual carbon and minerals, whereas incineration largely removes the carbonaceous fraction and forms mineral-rich SSA. This concentration of P and metals enhances resource-recovery potential but may also increase risks associated with heavy-metal accumulation, requiring the careful evaluation of composition, metal speciation, and leaching behavior.

The variability of SS composition is an important factor controlling thermochemical conversion and product quality. Differences in organic matter and ash content can substantially affect both the yield and properties of the resulting solid products. Pyrolysis of SS with a higher organic-matter content generally produces biochar with a greater degree of carbonization and aromatization, whereas differences in the initial heavy-metal content are reflected in the elemental composition of the resulting char [62,63,64]. A direct comparison of four SS feedstocks by Wang et al. further showed that biochar yield was positively correlated with the initial ash content, while higher organic-matter contents favored greater aromaticity, carbonization degree, and calorific value. Biochars produced from SS with higher initial heavy-metal contents also retained correspondingly higher metal concentrations, although their leachability generally decreased relative to the raw SS [62,63,64].

The inorganic composition of SS can additionally influence the textural and surface properties of SSC. Fan et al. showed that biochar derived from Al-rich SS exhibited a higher specific surface area, greater pore volume, stronger polarity, and a higher abundance of surface functional groups, indicating that mineral composition can affect both pore development and the retention of oxygen-containing functionalities during pyrolysis [62,63,64]. Consequently, variability in ash, organic matter, and mineral and heavy-metal contents can modify char yield, carbonization, pore development, surface chemistry, and the accessibility of catalytically active phases. Variations in N-, S-, and Cl-containing species are also important because they can influence heteroatom distribution in thermochemical products, downstream gas-cleaning requirements, and the stability of catalytically active mineral or metal phases.

### 3.2. Methods of SS Utilization

SS can be managed, recycled, or valorized through a range of routes selected according to product value, technological maturity, regulatory requirements, and process cost (Table A1). Current disposal and reuse routes include incineration (24.9%), agricultural use (49.2%), landfilling (8.7%), and land reclamation (12.4%) [65]. Incineration substantially reduces sludge volume but may generate toxic emissions, including HCN, and volatilized heavy-metal compounds such as Cd, Zn, As, and Pb [66]. Landfilling and agricultural use may result in the release of ecotoxic metals such as Cu, Zn, Cr, di(2-ethylhexyl)phthalate (DEHP) and polycyclic aromatic hydrocarbons (PAHs) [67,68,69]. Thermal and thermochemical treatment of SS can reduce certain environmental risks while enabling phosphorus recovery and the production of construction materials from the resulting residues. However, these routes may be constrained by contaminant content, emissions, resource quality, or increasingly stringent environmental regulations. In comparison, thermochemical processes such as pyrolysis, gasification, hydrothermal carbonization (HTC), and hydrothermal liquefaction (HTL) offer opportunities for energy and material recovery but remain less widely implemented at the commercial scale.

## 4. Thermochemical Plastic Co-Valorization with SS

With the continued development of waste-conversion technologies, thermochemical processes have emerged as promising routes for transforming SS into fuels, gaseous products, and carbonaceous materials. Table 6 summarizes the principal thermochemical methods applied to SS valorization. These processes differ substantially in the yields of gaseous, liquid, and solid fractions. However, process selection should also consider product composition, quality, upgrading requirements, and potential end use. Pyrolysis typically targets SSC and bio-oil, whereas gasification is primarily employed to maximize syngas production. HTC produces hydrochar as the main solid product, while HTL is generally designed to maximize the production of energy-dense biocrude.

### 4.1. Rationale for Integrating Sewage Sludge into Thermochemical Plastic Conversion

The integration of SS into thermochemical plastic conversion processes should not be regarded solely as the simultaneous treatment of two waste streams. The potential benefits of this approach arise from the complementary physicochemical characteristics of plastics and SS, as well as from interactions between their decomposition intermediates. Depending on the thermochemical conversion route, SS may perform different functions within the process. In co-pyrolysis and co-gasification, SS introduces oxygen- and nitrogen-containing organic compounds into the reaction system, while its mineral-rich inorganic fraction, containing Fe, Ca, Mg, Al, Si, and P, may exert catalytic effects and influence reaction pathways and product distribution [82,83]. In co-pyrolysis with plastics, improvements in both the yield and quality of the resulting oil have been reported, including the production of oils with higher heating values approaching those of conventional fuels [84,85]. In co-gasification, synergistic effects may be reflected in increased syngas yield and improved syngas quality, while the addition of plastic waste increases the higher heating value (HHV) of the feedstock mixture [86,87]. The moisture inherently present in SS can also participate in steam-reforming reactions and contribute to an increased hydrogen content in the resulting syngas [88,89]. Furthermore, SS offers the potential to integrate energy recovery with the recovery of mineral resources [90,91]. Depending on the selected conversion technology, these characteristics may influence reaction pathways, energy recovery efficiency, product distribution, and the management of inorganic residues.

However, the addition of SS does not necessarily improve plastic conversion performance under all conditions. The benefits of co-processing should therefore be evaluated against the separate conversion of both plastics and SS, rather than inferred solely from improvements relative to the treatment of SS alone. In particular, an increase in liquid or gaseous product yields may result from changes in the overall feedstock composition rather than from genuine synergistic interactions between the two feedstocks. Consequently, establishing the actual contribution of SS requires comparative analysis based on experimental data, including product yields and composition, energy recovery efficiency, and deviations from theoretically expected additive behavior.

In this review, a non-additive interaction is defined as a measurable deviation of the experimental response of an SS–plastic mixture from the value predicted by linear combination of the corresponding single-feed experiments conducted under comparable conditions. For a response variable Y, such as product yield or gas-component yield, the additive reference can be expressed as Equation (1):

(1) $$Y_{add}=w_{SS}Y_{SS}+w_{P}Y_{P}$$

where w_ss_ and w_p_ are the mass fractions of sewage sludge and plastic, respectively. The deviation from additive behavior is then expressed as Equation (2):

(2) $$\Delta Y=Y_{exp}-Y_{add}$$

or, where appropriate, as a normalized interaction index (in Equation (3)):

(3) $$S_{Y}=\frac{100\cdot (Y_{exp}-Y_{add})}{Y_{add}}$$

Similarly, thermogravimetric interactions can be evaluated by comparing experimental TG/DTG curves of the blend with curves calculated from the weighted contributions of the individual feedstocks [21,86,92]. Importantly, quantitative non-additivity demonstrates that the components do not behave independently, but does not by itself establish the underlying mechanism. Such deviations may originate from chemical interactions between volatile radicals, catalytic effects of the inherent mineral or carbonaceous fraction of SS, polymer melting and encapsulation, changes in interparticle heat and mass transfer, adsorption and secondary reactions on char, or changes in vapor residence time. Accordingly, the term “synergistic effect” is used here only for experimentally demonstrated beneficial deviations from an additive reference, whereas “non-additive interaction” is used when the existence of an interaction is established but its mechanistic origin has not been independently resolved. Negative deviations are described as inhibitory or antagonistic interactions.

The technological rationale for integrating SS with plastic waste also differs among conversion routes. Co-incineration can exploit the high calorific value of plastics to improve the energy efficiency of SS utilization [93], whereas in co-pyrolysis and co-gasification, additional effects may arise from interactions between plastic-derived radicals, SS decomposition intermediates, and catalytically active mineral components present in the sludge [82,94]. Hydrothermal processes enable the conversion of wet SS without requiring a preliminary drying step, although the conversion behavior of plastics under hydrothermal conditions strongly depends on polymer structure [95]. In addition, the use of SS-derived char and ash as catalysts represents an alternative integration strategy, in which the mineral and carbonaceous fractions derived from SS are utilized to upgrade plastic conversion products without directly introducing untreated SS into the feedstock [20,96].

Accordingly, the following subsections consider not only the applicability of individual thermochemical conversion technologies, but also the specific conditions under which the incorporation of SS provides measurable benefits or, conversely, introduces additional technological and environmental constraints.

To facilitate a direct comparison across the thermochemical conversion routes discussed below, Table A2 summarizes the principal differences between mono-conversion of plastics and SS and their integrated co-conversion, including the potential benefits and major limitations of each approach. Complementarily, Table A5 compiles experimental evidence from representative co-conversion studies, highlighting the investigated feedstock combinations, operating conditions, reference cases, and the experimentally observed effects of SS–plastic interactions. Together, these tables provide a comparative framework for distinguishing simple feedstock-blending effects from experimentally supported non-additive interactions.

### 4.2. Incineration

Incineration is a thermochemical treatment process in which combustible waste is oxidized under excess-air conditions to achieve near-complete conversion of its organic fraction. The principal products are flue gas and a solid ash residue. The flue gas consists mainly of CO_2_ and H_2_O, with possible emissions of NO_x_, SO_2_, CO, VOCs, and unburned hydrocarbons depending on feed composition and combustion efficiency. The solid ash is dominated by inorganic oxides but may contain residual carbon and volatiles [97,98]. Environmental concerns include gaseous pollutants and contaminant transfer to fly and bottom ash. Inadequate combustion or flue-gas treatment may release NO_x_, SO_x_, VOCs, BTEX, chlorinated organics, and other hazardous compounds [99]. Effective combustion control and gas cleaning are therefore essential.

#### 4.2.1. Incineration of Plastic Waste

The high carbon and hydrogen contents, low moisture, and high calorific values of plastics make them suitable for waste-to-energy (WtE) applications such as incineration (Table 7). PE and PP typically have heating values around 40 MJ·kg^−1^ or higher, comparable to liquid fossil fuels. PS also has a high energy content, although its aromatic structure can promote soot and PAH formation during incomplete combustion [100].

The environmental impact of plastic incineration depends strongly on polymer composition and combustion conditions. Because most conventional plastics are fossil-derived, their combustion releases fossil CO_2_, while incomplete oxidation can generate CO, VOCs, soot, and PAHs [100,101]. PVC is particularly problematic because of chlorine-containing emissions, while PVC and PET have been reported to produce higher VOC concentrations than several biodegradable plastics [99]. Additives and fillers may also transfer metals and other hazardous species to flue gas and ash. Unregulated burning can additionally generate toxic aerosols containing PAHs, PCDD/Fs, PCBs, and metal-containing particles [102].

**Table 7:** Main characteristics of polymers [103].

Plastic	PE	PP	PVC
Heat of combustion, MJ·kg^−1^	46.87	46.78	17.28–22.36
C	88.36	87.51	36.26–38.40
H	15.75	15.64	2.73–5.06
O	0.00	0.00	-
Cl	-	-	42.74–56.67

#### 4.2.2. Co-Incineration of SS with Plastics

**Figure 4:**
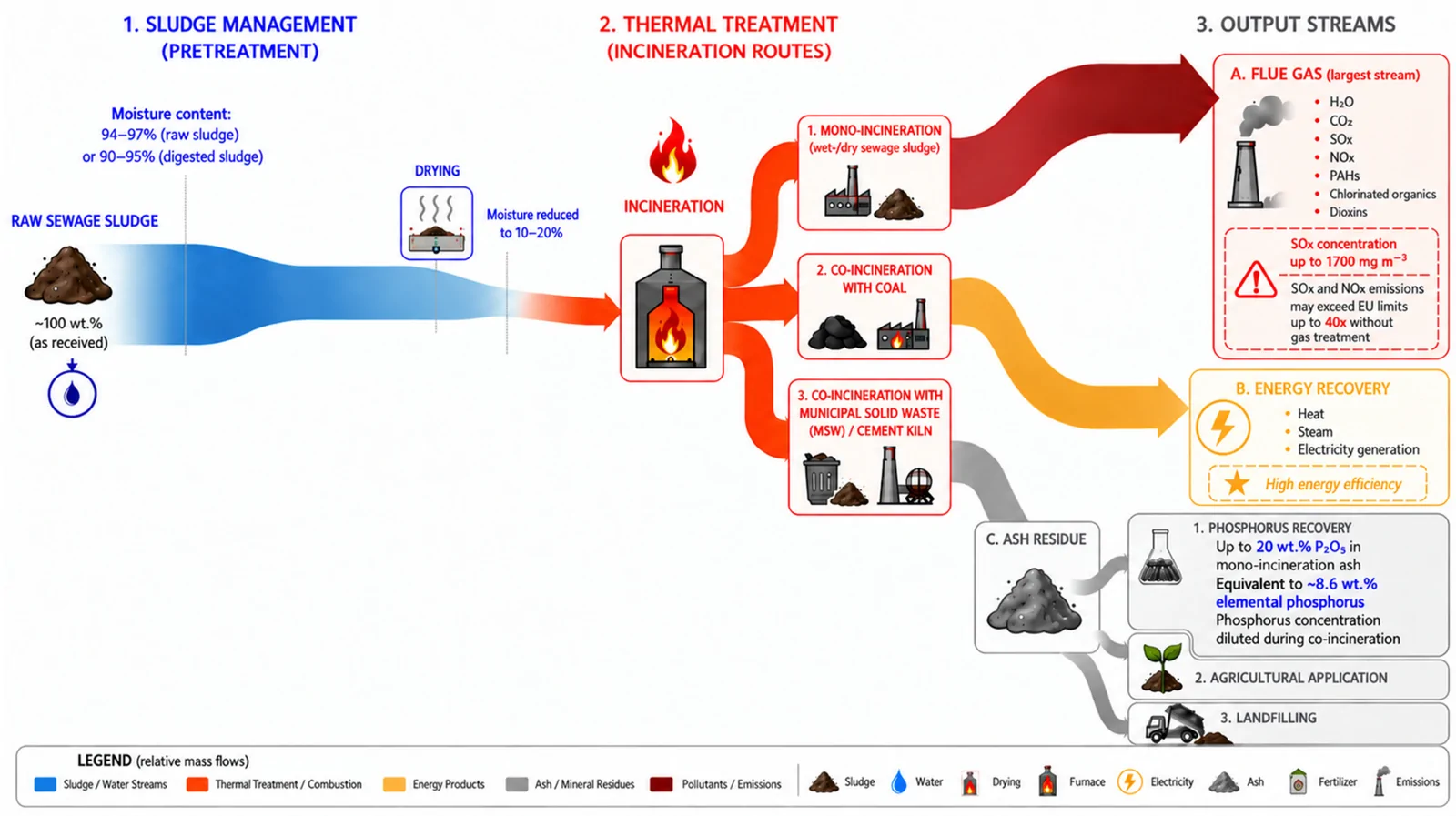
SS incineration route.

Co-incineration offers a complementary route for the utilization of plastic waste and SS. Figure 4 schematically depicts routes available for the incineration of SS, including SS preparation, mono-incineration, co-incineration with coal, and co-incineration with municipal solid waste including plastics. Plastics generally possess high volatile contents and heating values, whereas SS is characterized by high moisture and ash contents, relatively low heating value (LHV), and substantial concentrations of fuel-bound nitrogen, sulfur, chlorine, and metals. Plastic addition can therefore improve ignition, furnace temperature, and energy recovery, while SS may dilute chlorine-rich feeds and provide nitrogen- and sulfur-containing species that inhibit chlorinated organic pollutant formation. Early studies demonstrated this energetic complementarity. Park et al. (2008) estimated that a 50:50 mixture of agricultural plastic film and SS could provide a heating value of approximately 6073 kcal kg^−1^, although combustion was not experimentally investigated [104]. Lee and Bae (2009) examined SS pellets with plastic- and paper-containing refuse-derived fuel (RDF) in a laboratory stoker and found that sludge combustion slowed with increasing moisture and pellet diameter, whereas RDF addition substantially accelerated combustion [105].

Direct experiments with defined polymers further demonstrated the feasibility of co-incineration. Dąbrowski and Piecuch (2011) [106] investigated dried SS blended with PET, PVC, and PP. Increasing combustion temperature and excess air reduced CO emissions but generally increased NO_x_, while higher temperature also promoted SO_2_ formation. The high heating values of the plastics allowed fuel mixtures containing up to approximately 50% dry SS. Jamal et al. (2016) subsequently studied polystyrene–SS blends at 400–700 °C and found that increasing the plastic fraction enhanced the maximum combustion rate, although apparent activation energy also increased [107].

Pilot-scale investigations confirm the potential for self-sustaining operation. Zhu et al. (2013) [108] combusted pre-dried SS with PVC in a two-chamber screw incinerator. The much higher heating value of PVC (41.23 MJ kg^−1^) compared with SS (15.38 MJ kg^−1^) enabled self-sustaining combustion and secondary-chamber temperatures approaching 1000 °C. However, excessive plastic addition could promote melting, sticking, and agglomeration. Li and He (2013) [109] studied dried SS with PE in a 0.15 MW_th_ circulating fluidized-bed combustor. Increasing the SS contribution increased NO emissions because of its high nitrogen content, whereas increasing temperature strongly reduced N_2_O. Wet sludge produced substantially higher N_2_O than dried-SS/PE blends, emphasizing the importance of sludge drying.

Recent studies have extended the analysis to environmental performance. Jadlovec et al. (2025) [93] co-incinerated SS and predominantly PE automotive waste at a 1:1 ratio in a fluidized bed. PE substantially increased fuel LHV and energy recovery, although NO_x_ and SO_2_ remained important constraints. Ye et al. (2025) [110] investigated ternary combustion of SS, pine sawdust, and PE and showed that auxiliary fuels reduced burnout temperature and improved combustion indices. Although ash-fusion temperatures increased, significant slagging potential remained, while PE generally suppressed SO_2_ emissions.

Chlorinated plastics require particular attention. Gandon-Ros et al. (2021) [111] demonstrated that SS could act as an in situ inhibitor during PVC e-waste combustion at 850 °C. At a molar ratio of 0.75, PCDD/F formation was almost completely suppressed and dioxin-like PCB formation decreased by approximately 95%. Yan et al. (2012) similarly showed that nitrogen- and sulfur-containing gases released during sludge drying suppressed chlorobenzenes and PCDD/F formation [112].

Overall, the available evidence indicates that the main rationale for SS–plastic co-incineration is energetic and operational complementarity rather than a universal synergistic improvement in combustion performance. Plastic-rich feeds compensate for the LHV and high moisture and ash contents of SS by increasing fuel energy density, combustion rate, and furnace temperature, thereby facilitating stable or even self-sustaining operation [93,104,105,107,109]. However, this benefit is asymmetric: in most studies, plastics primarily improve the combustibility of SS, whereas the addition of SS does not inherently improve the energetic performance of plastic combustion. Therefore, increased heating value or enhanced burnout relative to SS mono-incineration should not by itself be interpreted as evidence of a true synergistic interaction.

The potential contribution of SS becomes more distinctive when environmental effects are considered. In chlorine-containing systems, sludge-derived nitrogen- and sulfur-containing species can suppress the formation of chlorobenzenes, PCDD/Fs, and dioxin-like PCBs, providing a functional benefit that cannot be explained solely by the higher calorific value of plastics [111,112]. Nevertheless, such advantages are strongly feedstock- and condition-dependent and may be offset by increased NO_x_/N_2_O formation, SO_2_ emissions, ash-related problems, slagging, heavy-metal redistribution, or PVC-derived HCl [93,109,110]. Thus, the benefit of co-incineration should be evaluated as a balance between improved thermal performance and additional emission and ash-management constraints. Current evidence supports co-incineration, particularly where low-energy SS requires a high-calorific co-fuel or where sludge-derived species can beneficially modify chlorine chemistry, but it does not demonstrate that SS addition is advantageous for all plastic types or combustion conditions. Systematic comparisons of plastic-only, SS-only, and mixed feeds under identical operating conditions are therefore still required to distinguish simple fuel blending effects from genuine process-level interactions.

### 4.3. Pyrolysis

Plastic pyrolysis converts polymers under oxygen-free conditions into pyrolysis oil (PyOil) or wax, pyrolysis gas (PyGas), and pyrolysis char (PyChar). Product distribution depends on polymer chemistry and operating conditions. PE and PP mainly undergo C–C scission, hydrogen transfer, and β-scission, producing alkanes, alkenes, waxes, and gases. PS readily depolymerizes to styrene and other aromatics, and PET undergoes ester cleavage, decarboxylation, and decarbonylation to oxygenated aromatics, CO, and CO_2_, whereas PVC first dehydrochlorinates at approximately 200–300 °C before further cracking and aromatization [113,114,115].

Temperature strongly controls conversion and secondary cracking. Moderate temperatures generally favor condensable products, while higher temperatures shift carbon toward gases, H_2_, aromatics, PAHs, and coke. Mixed LDPE/HDPE/PP produced more oil at 410 °C than at 550 °C [113]. For HDPE, increasing temperature and vapor residence between 500 and 900 °C enhanced cracking, H_2_ formation, PAHs, and coke [116], while LDPE at 700–1600 °C increasingly produced H_2_-rich gas and solid carbon [117].

Polymer structure modifies these trends. PS can give very high liquid yields, reaching 95.2 wt.% at 500 °C [118], while styrene represented 57–60% of major oil compounds in fixed-bed experiments [119]. PET instead favors gas and solid formation; at 550 °C it produced approximately 45.6 wt.% gas, 13.5 wt.% liquid, and 37.8 wt.% solid [120].

Heating rate, vapor residence, and heat transfer are interdependent. For HDPE at 650 °C, reducing vapor residence from 1.46 to 0.82 s increased oil from 68 to 80 wt.% [113]. Reactor design is therefore critical: fluidized beds provide better heat transfer and shorter vapor residence than fixed beds, increasing PS styrene yield from 64.5 to 78.7 wt.% [120]. Spouted-bed, auger, drop-tube, and microwave reactors further modify heat transfer and vapor history.

Overall, optimum pyrolysis requires matching polymer type, temperature, heating rate, vapor residence, reactor configuration, and quenching. PET and PVC additionally require management of oxygenated products and HCl, while mixed-plastic behavior can be non-additive [121].

#### 4.3.1. Catalysts Used in Thermochemical Conversion of Plastics

Catalysts are widely used in plastic pyrolysis to lower degradation temperature, accelerate cracking, and control the distribution of gases, liquids, and aromatics. Table A4 summarizes commonly applied materials. Catalytic performance is governed mainly by acidity, pore structure, surface area, and catalyst-to-plastic ratio. Zeolites are the most studied because their Brønsted and Lewis acid sites promote C–C scission, isomerization, hydrogen transfer, and aromatization. Common examples include HZSM-5, HUSY, HY, Hβ, mordenite, and FCC-type catalysts, while silica–alumina, mesoporous aluminosilicates, clays, metal oxides, and industrial residues provide alternative options [122,123].

For PE, strongly acidic HZSM-5 promotes cracking of long chains and waxes toward light gases, C_2_–C_4_ olefins, and monoaromatics, whereas larger-pore HUSY and Hβ can retain heavier liquids. Mesoporous MCM-41 and SBA-15 improve access of bulky intermediates, while MgO, ZnO, kaolin, Zn/SBA-15, and spent FCC offer lower-cost alternatives [123,124]. PP behaves similarly but is generally easier to crack because of its branched structure. Excessive acidity or catalyst loading, however, promotes over-cracking, gas formation, and coke [122,124].

For PS, catalysis primarily controls aromatic selectivity rather than conversion because thermal pyrolysis already yields styrene-rich liquids. Acidic zeolites promote conversion of styrene toward BTEX and polyaromatics, while CaO and some clays can better preserve styrene selectivity [123,125].

PET and PVC require different strategies. PET catalysts mainly promote deoxygenation or recovery of specific products such as benzoic acid, using materials including HZSM-5 and sulphated zirconia. PVC is more problematic because HCl release causes corrosion and catalyst poisoning; therefore, cracking catalysts such as Ni/ZSM-5 or Ni/SAPO-11 are often combined with chlorine sorbents such as Ca(OH)_2_ or red mud [126]. Overall, zeolites remain benchmark catalysts for PE, PP, and PS, while PET and PVC require simultaneous management of oxygen- and chlorine-containing products. Coke deposition remains a major deactivation mechanism, making pore structure, acid density, and regeneration critical for practical application [127].

#### 4.3.2. Co-Pyrolysis of Plastics with SS

**Figure 5:**
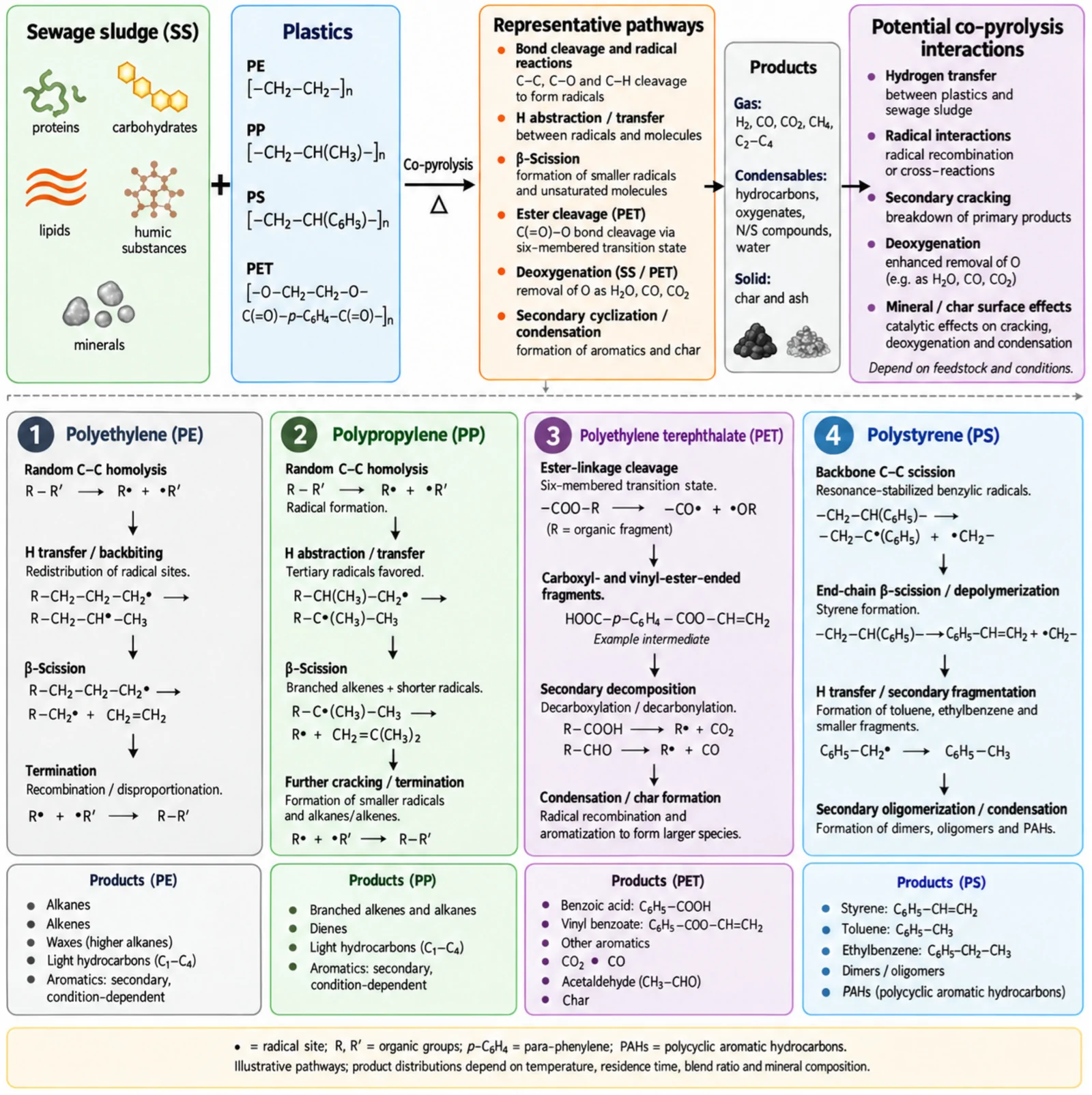
Schematic representation of PE, PP, PET, and PS co-pyrolysis reactions: illustrative pathways, product distributions depend on temperature, residence time, blend ratio, and mineral composition.

Co-pyrolysis of plastics with SS can result in non-additive conversion behavior arising from interactions between the two feedstocks. These interactions are commonly associated with hydrogen transfer between plastic-derived radicals and oxygenated or nitrogen-containing SS intermediates, but the observed deviation from additive behavior may also reflect catalytic effects of the inherent SS mineral phase, adsorption and secondary reactions on sludge-derived char, or changes in heat and mass transfer during blending [128,129]. Therefore, evidence of non-additivity and mechanistic attribution should be considered separately. Quantitative evidence is strongest where experimental behavior of the mixture is directly compared with a weighted additive reference derived from the corresponding single-feed experiments [21,92,128,129]. Figure 5 schematically depicts the co-pyrolysis reaction mechanisms. For SS–PP and SS–HDPE blends, Ling and Li confirmed synergism by TGA at 20, 50 and 80wt% plastic fractions and heating rates of 10–50 °C·min^−1^, where co-pyrolysis increased volatile conversion and reduced activation energy compared with individual feedstocks [129]. Quantitative kinetic evidence of non-additive behavior was reported by Chen and Huang for SS–PP co-pyrolysis [92]: using the Flynn–Wall–Ozawa method, the authors obtained mean apparent activation energies of 132.97, 183.83, and 207.01 kJ mol^−1^ for mixtures containing 10%, 30%, and 50% PP, respectively, compared with theoretical values of 292.84, 281.85, and 270.82 kJ mol^−1^. These correspond to reductions of approximately 54.6%, 34.8%, and 23.6%, calculated relative to the respective theoretical values. The comparison demonstrates a departure from the study’s additive kinetic reference, although it does not by itself establish the underlying mechanism or quantify a reduction in process energy consumption. For SS–LDPE, Zaker et al. showed that the main decomposition of the blend occurred in the 200–600 °C range, and the 1:1 SS:LDPE ratio was identified as optimal because it shifted the maximum temperature to a lower temperature and produced lower experimental char formation than the calculated additive value [21].

Quantitative comparison with additive reference behavior provides more direct evidence of non-additive interactions [21,92]. In SS–LDPE co-pyrolysis, the Coats–Redfern activation energy was 30.01 kJ·mol^−1^ for SS, 187.40 kJ·mol^−1^ for LDPE, and only 37.2 kJ·mol^−1^ for the 1:1 blend, showing that the blend behaved much closer to SS than to LDPE despite the high stability of polyethylene [21]. In the same work, iso-conversional methods gave activation energies of 200.37, 212.25 and 202.30 kJ·mol^−1^ by the KAS, FWO and Starink methods, while ΔH and ΔG for the 1:1 blend were 194.56–206.44 kJ·mol^−1^ and 119.28–119.54 kJ·mol^−1^, respectively [21]. In SS–PP co-pyrolysis, the actual FWO activation energy was far lower than the theoretical additive value: 132.97 vs. 292.84 kJ·mol^−1^ for 10% PP + 90%SS, 183.83 vs. 281.85 kJ·mol^−1^ for 30% PP + 70% SS, and 207.01 vs. 270.82 kJ·mol^−1^ for 50% PP + 50% SS [92]. These deviations quantitatively demonstrate that the blended feedstocks do not behave as an independent linear combination; however, reduced apparent activation energy alone does not distinguish radical-mediated chemical interactions from mineral catalysis or physical heat- and mass-transfer effects.

The type of plastic strongly controls the composition of the products [84,128]. For SS with HDPE and LDPE, Ma et al. found that the synergistic promotion effect for SS-HDPE was 190% higher than for SS–LDPE, indicating that polymer structure and density affect the release and interaction of radicals during decomposition. The same study reported that co-pyrolysis increased the hydrocarbon content of bio-oil by 2.7 times compared with SS mono-pyrolysis and reduced polluting gases such as NO_2_ and SO_2_. SS–LDPE favored short-chain hydrocarbons and naphthalene derivatives, whereas SS–HDPE favored longer chains and monocyclic aromatic hydrocarbons such as alkylbenzenes. The gasoline-range fraction in the oil reached 89.7% for the LDPE/SS mix and 55.8% for the HDPE/SS mix, showing that polyethylene addition can shift SS-derived oil toward lighter fuel-range hydrocarbons [128].

Polypropylene is also effective as a hydrogen-rich co-feedstock for SS because it contains almost 100% VM, has almost no ash, and has a heating value roughly three times higher than SS on a dry basis [92]. In SS–PP co-pyrolysis, 10% PP + 90% SS gave the maximum aromatic yield, while 30% PP improved the C5–12/C19+ carbon-number ratio and 10% PP gave the best overall pyrolysis-oil quality. The maximum C5–12/C19+ ratio was 9.1, and the optimal ratio of valuable petrochemical components, defined as alkenes + monoaromatics, to less desirable compounds, defined as polyaromatics + cyclic compounds, reached 4.02. Under the optimum oil-quality condition, the oil contained 19.72wt% alkanes, 61.58wt% alkenes, 7.65 wt% cycloaliphatics, 2.71wt% monoaromatics and 8.34wt% polyaromatics [92].

PET behaves differently from polyolefins because it is oxygen-rich and can form acidic aromatic products, but co-pyrolysis with SS can reduce some risks and improve char properties [130,131]. Qi et al. showed that the addition of SS reduced PET reaction activation energy by 78.83% and lowered the decomposition temperature by 8–30 °C in the 375–520 °C range and also reported that co-pyrolysis increased biochar yield by about 30% [131]. In microwave-assisted catalytic PET–SS co-pyrolysis, HZSM-5 increased bio-oil yield up to 49.5wt% and bioenergy density to 31.88 MJ·kg^−1^, while KOH promoted biochar yield up to 70.05wt% and improved fuel stability [130].

A more detailed mechanistic discrimination was recently provided by Ling et al., who investigated 50 wt% SS/PET co-pyrolysis using controlled sample configurations designed to distinguish vapor–vapor, vapor–solid, vapor–char, and mineral-mediated interactions. At 600 °C, the relative GC peak area of benzene increased from 1.78% for PET pyrolysis to 8.66% for the SS/PET mixture, while benzoic acid increased from 17.06 to 25.28% and 1,1′-biphenyl from 1.15 to 3.51%. In contrast, several higher-molecular-weight PET intermediates decreased, including dimethyl terephthalate from 3.72 to 1.64% and divinyl terephthalate from 16.62 to 9.55%. Benzaldehyde and acetophenone, accounting for 0.86 and 2.16% of the identified GC peak area, respectively, were detected only during co-pyrolysis and not during the separate pyrolysis of either feedstock. Complementary TGA–MS analysis also showed positive deviations of H_2_, CH_4_, H_2_O, CO_2_ and benzene evolution from the 50:50 weighted additive reference. Importantly, experiments separating SS and PET and using raw and demineralized SS-derived char indicated that vapor-phase interactions were a major origin of the observed non-additive product formation, while char-surface and inherent-mineral effects represented additional, distinguishable contributions [132].

Polystyrene is especially attractive for liquid-fuel production because it can form aromatic-rich oil, but catalytic upgrading is needed to control wax and improve product distribution. Xu et al. found that 60%PS + 40% dewatered sludge was the optimum composition and produced 11.21% higher energy recovery than the other tested compositions. With 3wt% ZSM-5, the upgraded PS-DS oil contained 49.24% aromatic hydrocarbons, 15.35% alkanes, 12.86% alkenes, 7.35% phenols and only 2.38% wax. The same study proposed an energy-recovery model capable of generating 20,863 kWh/day from 10 tons/day of biowaste, showing the potential of SS–PS catalytic co-pyrolysis for bio-circular energy systems [133].

The importance of transport effects is illustrated by Tomasek and Miskolczi, who compared thermogravimetric experiments using approximately 4 mg of feed with scaled-up tubular-reactor experiments using 4.0 g. Theoretical TG behavior and synthesis-gas yields were calculated from the weighted contributions of the individual feedstocks. In TGA, plastic addition was generally inhibitory, although the 25% SS + 75% plastic mixture exhibited a positive deviation from additive behavior of up to approximately 500 °C. In the scaled-up reactor, however, synthesis-gas yields exceeding the calculated additive values were observed for the 25% SS + 75% plastic and 50% SS + 50% plastic mixtures primarily at 900 °C. For the 25% SS + 75% plastic mixture, the H_2_/CO ratio reached 0.7 and the gas LHV was 23.6 MJ Nm^−3^ at 900 °C, compared with H_2_/CO ratios of 0.4 and 0.6 and LHVs of 17.6 and 20.3 MJ Nm^−3^ for SS and plastic alone, respectively. The authors attributed the shift in the beneficial interaction toward higher temperature at larger scale to poorer heat transfer and altered gas–solid and gas–gas interactions. These results demonstrate that experimentally observed synergy can be strongly scale- and transport-dependent rather than reflecting chemical interactions alone [134].

The environmental aspect of SS–plastic co-pyrolysis is complex because plastics can either reduce pollutant release or introduce additional heteroatoms such as Cl and N [84,135]. Shen et al. added 20% PP, PA6 or PVC to SS and pyrolyzed the mixtures at 800 °C for 60 min under N_2_, and the total mass loss increased from 54.58% for SS to 71.46% for PP+SS, 61.40% for PA6+SS and 64.04% for PVC+SS. PP, PA6 and PVC all inhibited NH_3_, CH_3_Cl, H_2_S and SO_2_ release, but PA6 increased HCN release and PVC increased HCl release because PA6 contains nitrogen and PVC contains chlorine. The potential ecological risk index decreased from 64.80 for raw SS to 26.57 for SS biochar, 26.84 for PP+SS biochar, 8.84 for PA6+SS biochar and 19.90 for PVC+SS biochar [84]. PVC-containing systems require special attention because chlorine can promote both heavy-metal volatilization and acid-gas formation [135,136]. In SS -PVC/CaO co-pyrolysis at 500, 700 and 900 °C, higher temperature and PVC addition reduced As, Pb and Zn contents in biochar, while CaO had the opposite effect by promoting immobilization. Cr, Cu and Ni were less volatile, and more than 90% of these metals remained in biochar under the tested temperature range. Metal-loaded PVC microplastics at SS:PVC ratios of 19:1, 3:1 and 1:3 increased Ca, Mg, Cd and Pb total concentrations in biochars but reduced the bioavailability of Cd, Cr, Ni and Zn [135].

Overall, co-pyrolysis currently provides the strongest experimental evidence among the reviewed thermochemical routes that combining SS with plastics can produce effects beyond simple feedstock blending. In particular, the deviations of experimental kinetic parameters and product formation from theoretically additive values in SS–LDPE, SS–PP and SS–HDPE systems demonstrate that interactions between the two feedstocks can modify decomposition pathways [21,92,129]. However, these effects are strongly polymer- and composition-dependent. Polyolefins primarily act as hydrogen-rich radical donors and can lower apparent kinetic barriers and shift SS-derived products toward lighter hydrocarbons [92,128,129], whereas PET and PVC introduce oxygen- and chlorine-containing reaction pathways that generate fundamentally different benefits and constraints [84,130,131,135,136]. Therefore, improvements observed for one polymer cannot be generalized to plastic waste as a single feedstock category.

Importantly, not all reported improvements constitute evidence that SS enhances plastic valorization. Several studies demonstrate better oil composition, energy recovery, or conversion relative to SS mono-pyrolysis, but such comparisons primarily show the beneficial effect of adding a hydrogen-rich plastic to sludge rather than a specific advantage provided by SS to plastic conversion [92,128,133]. Stronger evidence for a reciprocal benefit is obtained where SS lowers polymer decomposition barriers, modifies char formation, or alters theoretically expected product distributions [21,129,131]. Environmental performance is similarly non-uniform: PP can suppress several N-, S- and Cl-containing gaseous species, whereas PA6 and PVC introduce additional HCN and HCl, and PVC can modify heavy-metal volatilization [84,135,136]. Thus, the principal advantage of co-pyrolysis lies in the possibility of exploiting radical interactions and the inherent mineral phase of SS to modify conversion chemistry, but the direction and magnitude of this effect depend critically on polymer structure, SS/plastic ratio and operating conditions. Future comparisons should therefore prioritize experimentally measured deviations from additive behavior rather than improvements relative to SS alone. Representative quantitative evidence supporting these distinctions is summarized in Table A5.

#### 4.3.3. SS-Derived Catalysts for Pyrolysis

SS-derived catalysts are attractive because SS inherently contains both carbonaceous matter and mineral components such as Si, Al, Fe, Ca, Mg, and P. After pyrolysis, combustion, activation, or metal modification, these constituents can form catalytically active materials for cracking, deoxygenation, reforming, and aromatization. Metals accumulated in SS may provide intrinsic active sites, while the solid matrix can act as a catalyst support.

The pyrolysis of SS produces SSC, in which a carbonaceous matrix supports the mineral phase. In contrast, incineration and severe gasification largely remove this carbon matrix, producing mineral-dominated SSA. The carbonaceous fraction of SSC contributes porosity, adsorption sites, surface functional groups, and support for naturally occurring metallic species. SSC can therefore behave as a bifunctional catalyst, where the carbon matrix improves contact with pyrolysis vapors and the embedded minerals promote cracking, reforming, deoxygenation, and tar conversion. At elevated temperatures, however, degradation of the carbon matrix may contribute to coke formation [137].

The BET data summarized in Table 8 further demonstrate the favorable, but strongly temperature-dependent, surface characteristics of SSC. The porous carbon matrix facilitates molecular transport and access to adsorption and catalytic sites associated with surface functional groups and exposed Fe-, Ca-, Al-, Si-, Mg-, and P-containing phases. Kanafin et al. reported BET surface areas of 87.45 and 92.33 m^2^·g^−1^ at 500 and 700 °C, respectively, while the biochar produced at 900 °C exhibited the highest pore volume (0.101 cm^3^·g^−1^) and a well-developed mesoporous structure [138]. Similarly, Sierra et al. observed the highest specific surface area (113 m^2^·g^−1^) and pore volume (0.156 cm^3^·g^−1^) at 800 °C, whereas treatment above 900 °C substantially deteriorated the textural properties due to pore deformation, cracking, or blockage [139]. Although these values remain lower than those of HZSM-5 (350–450 m^2^·g^−1^, 0.12–0.30 cm^3^·g^−1^) [140], Zeolite Y (600–900 m^2^·g^−1^, 0.25–0.45 cm^3^·g^−1^) [141] and activated carbon (1000–2000 m^2^·g^−1^, 1.19–2.20 cm^3^·g^−1^) [142], the catalytic potential of SSC cannot be assessed from BET surface area alone, because pore accessibility, surface functional groups, and exposed mineral phases also determine the availability of active sites. Chemical analysis of biochar is shown in Table 9

The catalytic properties of SSC are strongly influenced by its mineral composition, particularly its metal-containing phases. Metals such as Ni, Pt, Pd, Fe, Co, Zn, Cu, and Mn are known to promote cracking and related reactions [143]. As shown in Table 9, Al_2_O_3_ can provide acidic sites, whereas MgO and CaO mainly contribute basic sites. Iron-containing phases are especially important because they may promote secondary cracking, reforming, and dehydrogenation of pyrolysis vapors, thereby reducing tar and increasing gases such as H_2_, CO, and CH_4_. However, catalytic activity depends strongly on Fe speciation and accessibility. During pyrolysis, Fe may transform into reduced oxides, metallic Fe, sulfides, or phosphate/silicate phases with different activities. Therefore, bulk Fe_2_O_3_ content alone is insufficient to predict performance and should be interpreted together with XRD, XPS, or Mössbauer analysis [144,145,146,147].

Sun et al. demonstrated that SSC can strongly promote aromatization during plastic pyrolysis. At 600 °C, monocyclic aromatics reached 75.3%, whereas at 800 °C bicyclic aromatics reached 64%. The authors attributed this behavior to P=O and sulfide groups acting as dehydrogenation centers, while CaO- and Fe-containing phases promoted ring-opening reactions contributing to aromatic formation [20]. Zhou et al. similarly showed that SSC can enhance steam reforming, with the carbon matrix providing catalytic support and Fe_2_O_3_ cracking functionality for H_2_ production, although aromatic compounds remained abundant in tar and liquid products [148].

The catalytic potential of SSA is less extensively studied. Because incineration removes most of the carbonaceous matrix, SSA is dominated by mineral phases and generally has a lower specific surface area than SSC. Nevertheless, Gao et al. reported that increasing SSA addition during MSW pyrolysis raised gas yield from 65.5% to 73.2–76.4%, increased longer-chain hydrocarbons and H_2_ in the gas by 5–10%, and reduced liquid yield from 20.8% to 6.4% [149]. Lin et al. also showed that SSA can promote deoxygenation during pyrolysis of photosensitive resin waste and polycaprolactone waste, with ester formation differing by about 20% compared with Al_2_O_3_ and Fe_2_O_3_ [96].

The catalytic properties of SSC and SSA can be improved through physical or chemical activation. NaOH treatment increased SSA surface area from 0.92 to 8.11 m^2^·g^−1^ and pore volume from 0.01 to 0.04 cm^3^·g^−1^ [150]. Latosińska et al. converted SSA into zeolite P using NaOH treatment, calcination, and crystallization. Lower crystallization temperatures produced higher surface areas, whereas higher temperatures increased Al and Si incorporation, cation-exchange capacity, and zeolitization [151,152,153]. H_2_SO_4_ activation similarly increased SSA surface area from 1.51 to 16.18 m^2^·g^−1^ [154].

Activation of SS-derived supports can target not only the mineral phase but also the carbon matrix. Wu et al. pyrolyzed waste-activated sludge and treated the product with HCl, HNO_3_, or NaOH. Specific surface area increased from 11.64 m^2^·g^−1^ to 179.81 m^2^·g^−1^ after NaOH treatment and 177.59 m^2^·g^−1^ after HNO_3_ treatment, accompanied by a shift from mesoporous toward microporous structure, making the products more comparable to sludge-derived activated carbon than ash-derived zeolites. Chemical treatment also increased C–O and –OH surface groups, providing potential acidic or anchoring sites for supported catalysts [155]. Other activators include HF, amines, and ZnCl_2_. Reported surface areas were 38.63 m^2^·g^−1^ after ZnCl_2_ activation, 101.23 m^2^·g^−1^ after NaOH treatment, and 50.69 m^2^·g^−1^ after HF treatment. HF produced pores of approximately 9 nm versus about 5 nm after NaOH treatment, suggesting pore widening or structural damage [156]. Mild oxidation can further functionalize the surface. Atienza-Martínez et al. treated anaerobically digested SS with air at 200 °C and reported an approximately 6% increase in C–O and C=O groups [157,158].

Metal loading is a major strategy for increasing the activity of SS-derived catalysts toward pyrolysis-vapor upgrading, tar cracking, and reforming. Native Fe-containing phases and acidic Al_2_O_3_–SiO_2_ sites can participate directly, whereas additional Ni, Fe, Co, Mn, or bimetallic species can enhance cracking, reforming, dehydrogenation, and aromatization. In steam gasification of SS using Ni-loaded SSC, the best performance was reported at 900 °C, where gas yield increased from 0.02 to 0.76 m^3^·kg^−1^. A Ni loading of 20% was optimal, while higher loading promoted pore blockage and coking [159].

Gao et al. impregnated SS fly ash with Co and applied it in a two-stage fixed-bed pyrolysis/reforming system. Relative to non-catalytic co-pyrolysis, the catalyst decreased CO yield by about 5%, increased H_2_ yield by approximately 10%, and raised total gas yield by roughly 10%, mainly by converting condensable vapors into gaseous products [150]. Liu et al. used biochar derived from municipal SS and cotton stalks, followed by KOH activation and Ni/Ca functionalization. H_2_ yield increased from 3.73 mmol·g^−1^ with quartz sand to 3.94 mmol·g^−1^ with raw biochar and 4.00 mmol·g^−1^ after KOH activation. Ni and Ca increased H_2_ to 4.42 and 4.33 mmol·g^−1^, respectively, whereas Ni/Ca reached 5.03 mmol·g^−1^. Total gas yield rose from about 7.03 to 8.89 mmol·g^−1^, with Ni/Ca producing 5.03 mmol·g^−1^ H_2_, 2.14 mmol·g^−1^ CO, 1.04 mmol·g^−1^ CO_2_, and 0.68 mmol·g^−1^ CH_4_. This improvement was attributed to Ni-assisted tar cracking, steam reforming, and water–gas shift reactions, while CaO promoted CO_2_ capture and shifted equilibrium toward H_2_ formation [160].

Bimetallic catalysts can also improve stability and suppress coking. Daorattanachai et al. found that a Re ratio of 3:7 at 900 °C gave the best conversion and H_2_ yield during biomass cracking. Coke deposition decreased from 15.6 mmol·g^−1^ catalyst for Ni/SSC to 5.6 mmol·g^−1^ for 3:7 Re–Ni/SSC, with stability maintained for 18 h [161]. Wang et al. used municipal sludge char as a support for Fe-, Ni-, and Fe–Ni-based catalysts in toluene reforming. Bare sludge char achieved 42.6% conversion, whereas Fe/MSC, Ni/MSC, and Fe–Ni/MSC reached 90.5%, 92.1%, and 92.6%, respectively. Fe–Ni/MSC also gave the highest H_2_ selectivity at 73.6 mol.%, attributed to active phases including Fe_2_SiO_4_, FeO(OH), NiSi, Fe_3_O_4_, and NiFe_2_O_4_ [162].

Overall, the available studies show that the value of SS-derived materials in plastic pyrolysis does not arise from their ability to reproduce the properties of conventional zeolites, but from their combination of low-cost mineral functionality, waste-derived carbon support, and potential for further chemical modification. Unmodified SSA and SSC generally possess substantially lower surface areas than commercial zeolites or activated carbons, indicating that intrinsic porosity alone cannot explain their catalytic performance [143,144,145,146]. Instead, activity is associated with the combined effects of Fe-, Ca-, Mg-, Al- and Si-containing phases, while SSC provides an additional carbonaceous matrix capable of adsorption and secondary surface reactions. Consequently, SSA should primarily be regarded as a mineral catalyst, whereas SSC behaves as a more complex bifunctional material whose carbon and inorganic fractions act simultaneously.

However, catalytic performance cannot be predicted reliably from bulk elemental composition alone. The examples summarized in this section demonstrate that activation and metal loading can markedly improve H_2_ formation, tar conversion and catalyst stability [150,155,159,160,161], but they also show that increasing metal loading or reaction severity can promote pore blockage, coke formation and loss of activity. Particularly for Fe-containing materials, mineral speciation and accessibility are more informative than total Fe concentration [143,144,145,146]. Moreover, many reported studies concern model tar compounds, biomass-derived vapors or heterogeneous waste rather than isolated plastic feedstocks, limiting direct comparison with conventional plastic-pyrolysis catalysts. Therefore, SS-derived catalysts are most convincingly justified as a resource-recovery strategy that simultaneously valorizes sludge solids and reduces dependence on virgin catalytic materials, rather than as universal replacements for zeolites. Direct benchmarking against commercial catalysts under identical plastic-pyrolysis conditions, including activity, selectivity, deactivation and regeneration, remains necessary.

#### 4.3.4. Pyrolysis Reaction Mechanisms in the Presence of SSA and SSC

The catalytic behavior of SSA and SSC during plastic pyrolysis depends strongly on polymer structure and on the distinct physicochemical properties of the two materials. SSA consists predominantly of inorganic mineral phases containing Si, Al, Fe, Ca, Mg, and P, whereas SSC retains a carbonaceous porous matrix together with these mineral species. Consequently, SSA acts primarily through mineral acid–base and redox sites, while SSC combines adsorption and surface reactions on carbon with catalysis by embedded inorganic phases.

**Figure 6:**
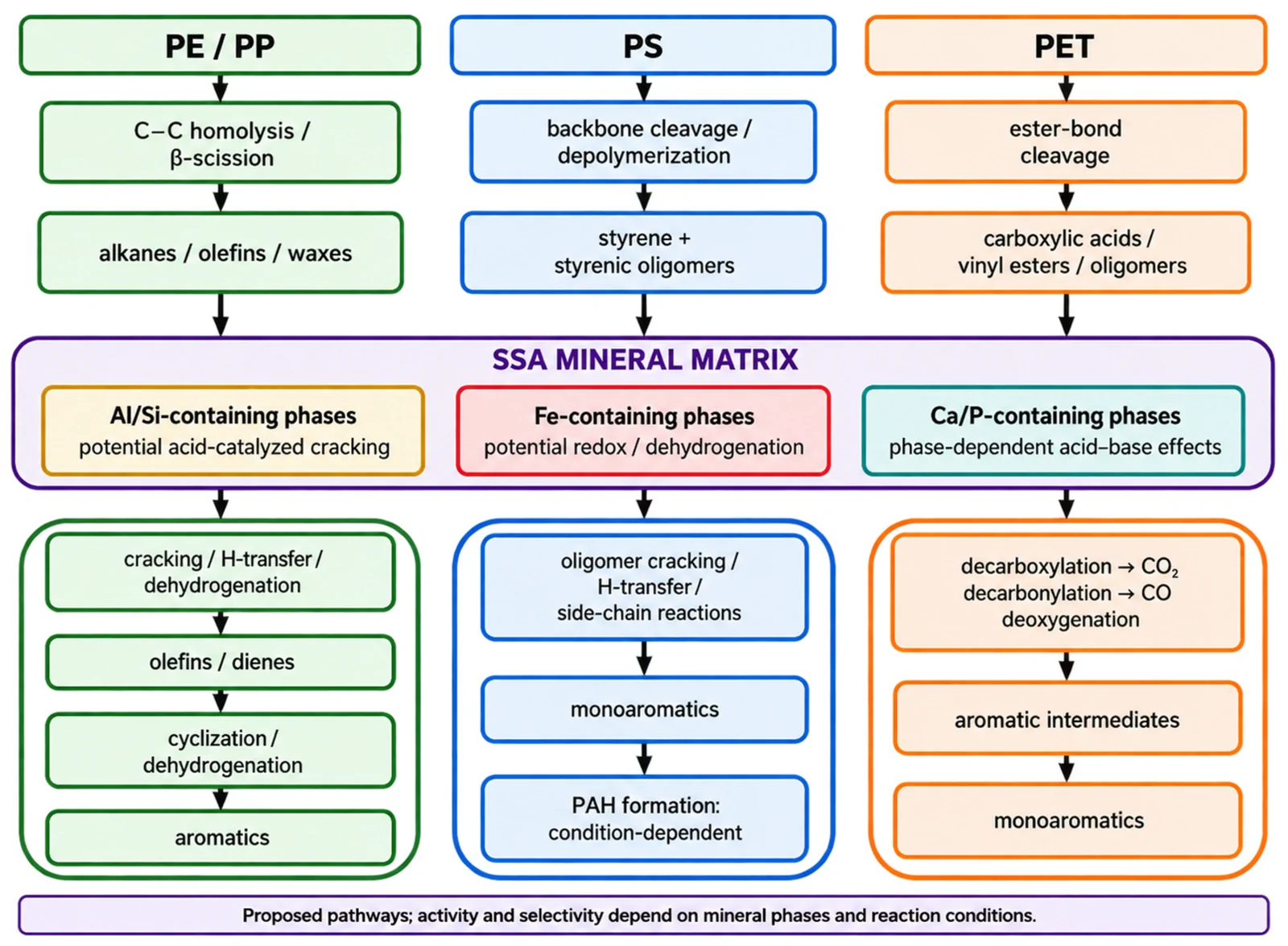
PE, PP, PS, and PET pyrolysis mechanism with SSA as a catalytic material.

Figure 6 schematically depicts reaction mechanisms occurring during pyrolysis of PE, PP, PS, and PET with SSA used as a catalyst. For PE and PP, thermal pyrolysis initially proceeds through C–C homolysis, radical formation, and β-scission, yielding olefins, dienes, paraffins, and waxes. Over SSA, Al- and Fe-containing phases can promote secondary cracking and dehydrogenation, while Ca/Mg-containing basic sites may facilitate hydrogen-transfer and rearrangement reactions. Fe oxides can also participate in redox transformations. Fe_2_O_3_ has shown to undergo partial reduction during catalytic plastic pyrolysis, indicating the direct participation of iron species in secondary reactions. These pathways increase the pool of short olefins and dienes that can undergo cyclization and subsequent dehydrogenation to aromatics. PP is generally more favorable for this route because β-scission readily forms branched and substituted olefins, whereas PE produces more linear wax-like intermediates and therefore retains a stronger competing cracking pathway. Lin et al. demonstrated that sludge ash and its Fe/Al oxide components influence depolymerization, dehydrogenation, cyclization, and deoxygenation reactions during plastic pyrolysis.

PS differs because its phenyl rings are already incorporated into the polymer backbone. Pyrolysis therefore generates styrene, benzyl radicals, and styrenic oligomers rather than requiring de novo aromatic-ring formation. SSA mainly promotes secondary cracking, side-chain transformation, and hydrogen-transfer reactions, shifting the distribution among styrene, alkylbenzenes, lighter monoaromatics, and condensed PAHs. Thus, its role is better described as aromatic redistribution than aromatization.

**Figure 7:**
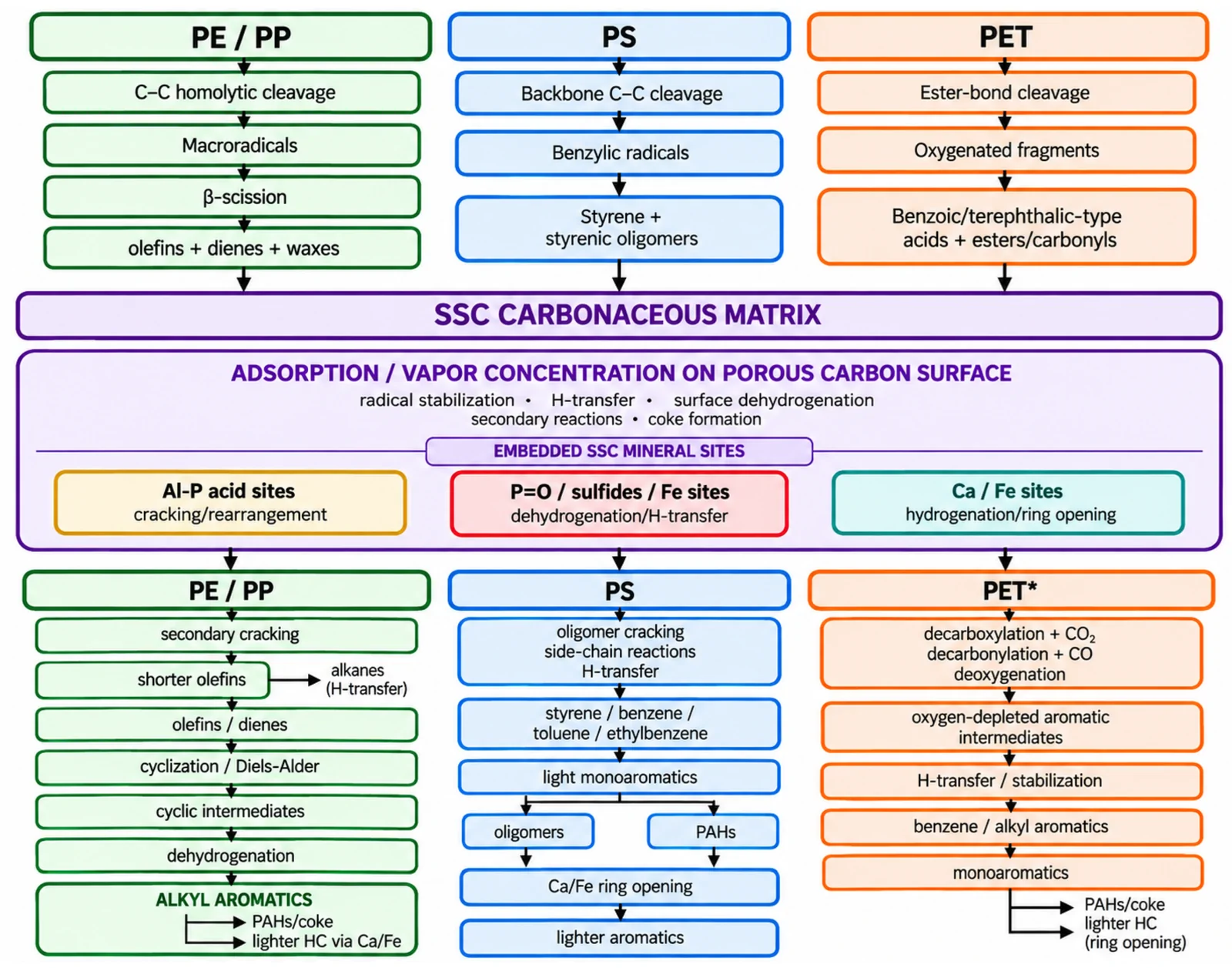
PE, PP, PS, and PET pyrolysis mechanism with SSC as a catalytic material. * PET-SSC catalytic steps are proposed/inferred; direct mechanistic evidence for isolated PET pyrolysis over pre-produced SSC is currently limited.

Figure 7 schematically depicts reaction mechanisms occurring during the pyrolysis of PE, PP, PS, and PET with SSC used as a catalyst. SSC introduces an additional carbon-mediated pathway. Plastic-derived vapors can adsorb on the porous carbon matrix, where radical stabilization, hydrogen transfer, surface dehydrogenation, and secondary condensation occur before or simultaneously with reactions on mineral sites. Sun et al. showed that Al-, P-, and S-containing species increase SSC acidity, while P-, Fe-, and S-containing sites promote dehydrogenation and cyclization. CaO and Fe species suppress excessive aromatic condensation through hydrogenation and ring-opening. Accordingly, SSC promotes cracking–dehydrogenation–cyclization of PE/PP intermediates, while for PS it primarily redistributes styrenic compounds between light aromatics, oligomers, and PAHs.

For PET, the dominant catalytic objective is deoxygenation. PET first forms terephthalate-, benzoic-acid-, ester-, and carbonyl-derived intermediates. SSA can promote C–O cleavage, decarboxylation, and decarbonylation, producing CO_2_, CO, and lower-oxygen aromatic products. Fe_2_O_3_ has specifically been shown to suppress acidic products through decarboxylation. For SSC, the carbon matrix may additionally adsorb on and stabilize oxygenated aromatic radicals, while embedded Fe/Ca/Al species promote oxygen removal and secondary aromatic conversion. However, direct mechanistic studies of isolated PET pyrolysis over pre-produced SSC remain scarce. Therefore, the PET–SSC pathway should presently be regarded as mechanistically plausible rather than conclusively established.

**Figure 8:**
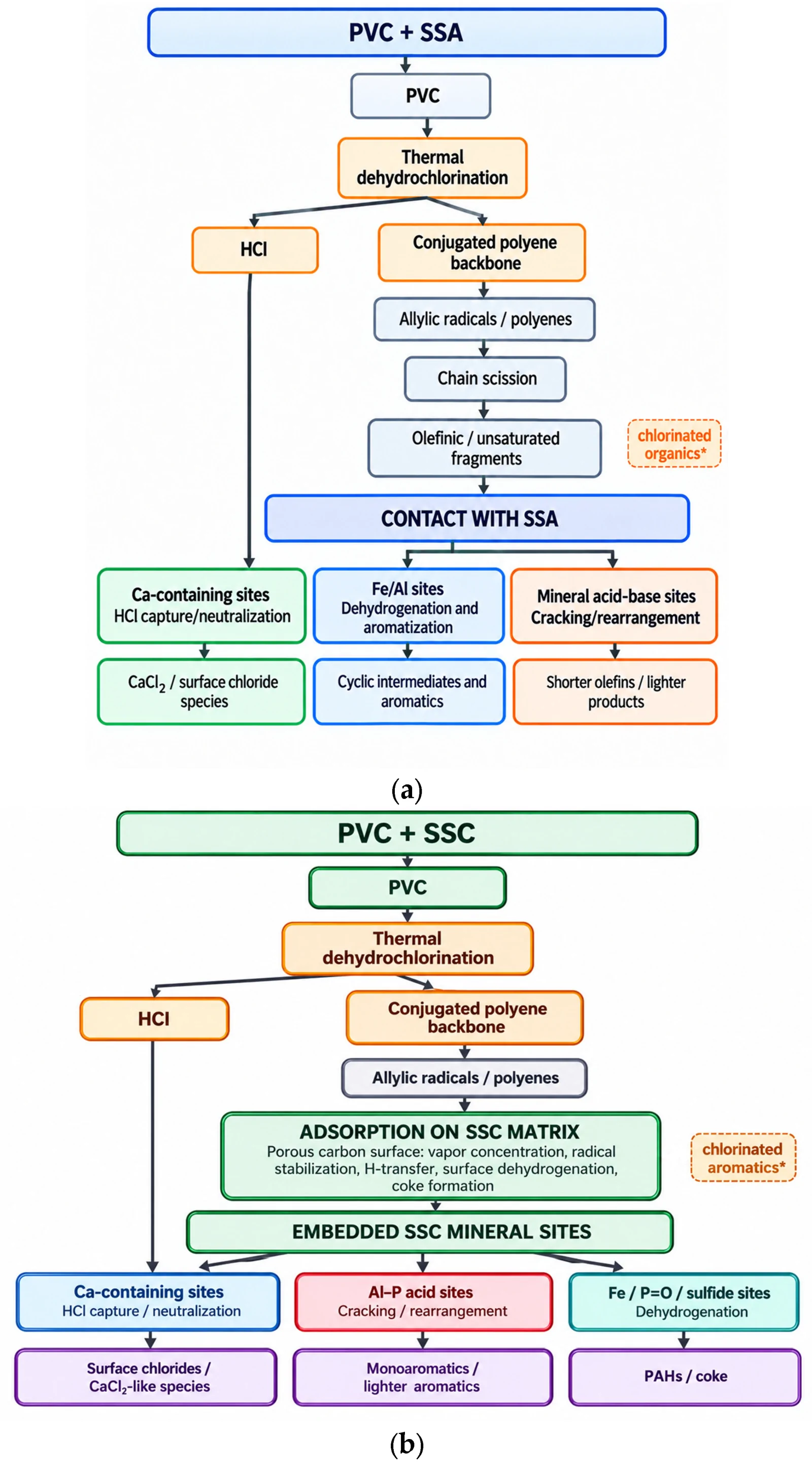
PVC pyrolysis mechanism with (**a**) SSA as a catalytic material and (**b**) SSC as a catalytic material. * Chlorinated aromatics deposit on SSC.

PVC follows a distinct pyrolysis pathway compared with other plastics because dehydrochlorination dominates its early thermal decomposition as shown in Figure 8. PVC first undergoes successive C–Cl and C–H bond cleavage at relatively low temperatures, releasing HCl and forming conjugated polyene chains. During SS–PVC co-pyrolysis, major mass loss occurs at 160–400 °C due to dehydrochlorination and chlorinated species release, while at 400–550 °C polyenes undergo cyclization and secondary cracking to yield benzene and other aromatic compounds. The high HCl yield makes chlorine management a key issue in catalytic systems.

In SSA, PVC conversion is mainly driven by heterogeneous mineral catalysis. After dehydrochlorination, polyene fragments interact with Fe-, Al-, Ca-, Si-, and P-containing phases. These minerals can promote chain scission, dehydrogenation, cyclization, and aromatization. Ca-containing species also capture HCl, forming inorganic chlorides and reducing corrosion, although efficiency depends on mineral form and temperature. The remaining carbon skeleton is further converted into monoaromatics and PAHs, with continued condensation leading to coke formation. However, The detailed mechanistic roles of individual SSA components remain insufficiently established.

SSC provides an additional carbon-based reaction environment. Its porous carbon matrix enables the adsorption of PVC-derived HCl, polyenes, and aromatic vapors, while embedded minerals catalyze cracking and hydrogen-transfer reactions. SSC can therefore enhance secondary decomposition and aromatization. However, HCl also deactivates SSC by forming metal chlorides that block pores and damage structure, reducing catalytic performance.

Chlorine transfer to inorganic metals is another important pathway. Released chlorine reacts with sludge-derived metals to form metal chlorides, altering catalyst composition and potentially increasing metal volatilization due to their low melting points.

Overall, PVC pyrolysis over SSA and SSC proceeds through dehydrochlorination followed by polyene cyclization and aromatization. SSA mainly promotes mineral-catalyzed HCl capture and hydrocarbon conversion, while SSC additionally provides carbon-surface reactions but is more vulnerable to chloride-induced deactivation.

Taken together, the proposed mechanisms indicate that the catalytic role of SS-derived materials is strongly controlled by both polymer structure and the physical form of the sludge-derived catalyst. For PE and PP, the most plausible contribution of SSA and SSC is enhancement of secondary cracking, dehydrogenation and cyclization of radical-derived intermediates, whereas for PS the dominant effect is redistribution of already aromatic styrenic products rather than formation of aromatic rings de novo. PET and PVC require different catalytic functions because oxygen and chlorine removal become central to product upgrading. These distinctions show that a single generalized “SS-derived catalyst mechanism” is not appropriate for all polymers.

At the same time, the mechanistic evidence is uneven. Reactions involving mineral oxides, Fe-containing phases and acid–base sites are supported by experimental trends and studies of individual catalyst components, whereas several pathways involving adsorption, radical stabilization and secondary reactions on the SSC carbon matrix remain inferred from catalyst properties and product distributions rather than directly observed. This limitation is particularly important for PET–SSC, for which direct mechanistic studies remain scarce, and for PVC, where simultaneous HCl capture, metal chlorination and catalyst deactivation complicate interpretation. Therefore, the reaction schemes in Figure 6 and Figure 7 should be regarded as evidence-based mechanistic frameworks rather than fully established elementary reaction networks. Future work combining isotopic tracing, in situ spectroscopy, phase-resolved catalyst characterization and product-specific kinetic analysis would be required to distinguish the respective contributions of carbon surfaces and individual mineral phases.

### 4.4. Gasification

Plastic gasification is a thermochemical process in which polymers are partially oxidized at temperatures typically above 600 °C using controlled amounts of air, O_2_, steam, CO_2_, or their mixtures. The main product is syngas containing H_2_, CO, CO_2_, CH_4_, and light hydrocarbons. Unlike pyrolysis, which generally targets liquids and waxes, gasification aims to maximize conversion to gaseous products for heat, power, H_2_, methanol, or chemical synthesis [163,164,165]. It is particularly suitable for heterogeneous or contaminated plastic waste.

The gasifying agent strongly affects syngas quality. Air gasification can operate autothermally but produces N_2_-diluted gas with heating values around 6–8 MJ·m^−3^, whereas steam gasification yields N_2_-free, H_2_-rich gas exceeding 15 MJ·m^−3^ but requires external heat [165]. O_2_-, steam/O_2_-, CO_2-_, and supercritical-water gasification have also been investigated [166].

Reactor configuration is important because molten plastics have low thermal conductivity and high viscosity. Fluidized beds provide efficient heat and mass transfer and good temperature control [165,167], while spouted beds facilitate handling of sticky feeds. Pilot-scale bubbling fluidized-bed gasification has been demonstrated at capacities up to 100 kg·h^−1^ [168]. Olivine can additionally promote tar conversion and produce approximately 20–30% H_2_ from polyolefin-rich feeds [169].

Temperature and equivalence ratio control gas yield and tar formation. Increasing temperature from 600 to 900 °C enhanced H_2_, CO, and CH_4_ while reducing tar; optimum performance was reported near 800 °C and an equivalence ratio of 0.25 [170]. Tar remains a major challenge, encouraging the use of dolomite, olivine, Ni-, Fe-, and Co-based catalysts or two-stage pyrolysis–steam reforming for the production of H_2_-rich, low-tar syngas [164,165].

#### Co-Gasification of SS with Plastics

Co-gasification of SS with waste plastics is an emerging thermochemical route that combines two feedstocks with highly complementary properties. SS typically has a high ash content and relatively low carbon and hydrogen contents, whereas plastics such as PE, PP and PS are highly volatile, carbon- and hydrogen-rich materials with little mineral matter. Consequently, introducing plastics can increase the energy density and hydrogen availability of an SS-based gasification feed, while the moisture, oxygenated products and inherent mineral matter originating from sludge may contribute to steam reforming and secondary cracking reactions. Nevertheless, the literature directly investigating SS–plastic co-gasification remains very limited compared with the much larger body of work on co-pyrolysis.

The most comprehensive direct study was reported by Yang et al., who investigated SS mixed with PE, PP or PS at plastic fractions of 25, 50 and 75 wt%. The feed was first converted at 600 °C, and the resulting vapors subsequently passed through a steam-reforming zone operated between 700 and 900 °C. Increasing the secondary temperature enhanced syngas formation and, particularly, H_2_ production, while simultaneously decreasing the mass yield of tar. Plastic addition shifted the gas composition from the more oxygenated products characteristic of sludge toward H_2_, CH_4_ and hydrocarbon gases. Among the investigated plastics, PE produced the strongest increase in H_2_. Synergistic analysis further showed that experimentally measured H_2_ and CH_4_ yields exceeded the values predicted from simple additive behavior, whereas the yield of heavier gaseous hydrocarbons, expressed as C_n_H_m_, was lower than predicted. This indicates intensified secondary cracking of larger hydrocarbon intermediates during co-gasification [86]. The influence on tar was particularly significant. Increasing the secondary gasification temperature shifted carbon from the condensable fraction into syngas. At 900 °C, the reported tar concentration from SS alone was 239 g Nm^−3^, compared with 51.6, 97.5 and 100.1 g Nm^−3^ for PE, PP and PS, respectively, on a dry-gas basis. Thus, under identical conditions, sludge generated approximately two to four times more tar than the individual plastics. The authors attributed SS tar to a chemically complex mixture containing aldehydes, alcohols, ketones, phenols and other oxygenated organic compounds, which progressively decomposed into H_2_, hydrocarbons, CO and CO_2_ as the secondary temperature increased [86]. Plastic addition also altered the nature of the condensable intermediates. During co-gasification, radicals produced from plastic decomposition promoted the conversion of SS-derived phenols, furans, alcohols and ketones into lighter products. The inorganic constituents of SS, including alkali, alkaline-earth and transition-metal species, may additionally contribute to catalytic cracking. However, increasing the PP or PS fraction beyond approximately 50 wt% reduced overall gasification efficiency because larger quantities of relatively stable C_12_–C_20_ hydrocarbons and C_21+_ waxes were formed and could not be completely cracked under the selected conditions. Accordingly, at 800 °C, approximately 50 wt% PP or PS represented the optimum investigated composition, whereas gasification efficiency continued to increase with increasing PE content.

Importantly, Yang et al. quantified these interactions rather than inferring synergy solely from improved gasification performance. The interaction coefficient ƒ, calculated from deviations between experimental and additive thermogravimetric behavior, varied with both polymer type and reaction stage [86]. At 50 wt% plastic, ƒ_1_ values were 1.21, 1.02 and 1.15 for PE, PP and PS, respectively, whereas the corresponding ƒ_2_ values were 0.85, 1.29 and 1.22. Thus, the same SS–plastic combination may shift between promoting and inhibiting behavior during different conversion stages. In the two-stage reactor, experimentally measured H_2_ and CH_4_ yields were also higher than the weighted values calculated from the individual feedstocks, whereas C_n_H_m_ yields were lower, indicating enhanced secondary conversion of larger hydrocarbon intermediates [86]. Nevertheless, these quantitative deviations should not be interpreted exclusively as chemical synergy. Yang et al. identified several competing contributions: melted polyolefins can encapsulate SS particles and increase heat- and mass-transfer resistance, while the same molten phase can prolong the contact of SS-derived volatiles with reactive intermediates; in parallel, alkali, alkaline-earth and transition-metal species in SS may catalyze decomposition and secondary cracking [86]. The measured non-additivity therefore represents the net result of chemical, catalytic and transport-related interactions.

The work of Li et al. provides further insight into the interaction between SS-derived vapors and plastic conversion. In their staged system, SS was pyrolyzed at 450 °C and the generated steam, gases and condensable vapors subsequently interacted with PP at 600–800 °C. This configuration differs from conventional simultaneous co-gasification but directly demonstrates how SS-derived products can affect downstream plastic conversion [171]. A particularly interesting temperature-dependent behavior was observed. At a PP conversion temperature of 700 °C, the coupled process produced more condensable liquid and less gas than predicted from the independent conversion of the two feedstocks, indicating incomplete secondary cracking. At 800 °C, however, this behavior reversed: condensable yield decreased and gas formation increased markedly, demonstrating effective conversion of liquid/tar intermediates into gaseous products [171]. This illustrates an important general phenomenon in gasification: tar quantity and tar quality may evolve in opposite directions. Thermal cracking can substantially reduce total tar while simultaneously converting surviving compounds into increasingly condensed and refractory aromatic structures. The pilot-scale study of Lin et al. is also relevant because the simulated municipal waste contained both PP and PE together with SS and lignocellulosic material. A two-stage fluidized-bed configuration was used, with activated carbon and Ni- or Fe-modified activated carbon in the secondary stage. Increasing steam addition promoted reactions associated with hydrocarbon and tar reforming, while Ni/AC was more effective for H_2_ production than Fe/AC. The maximum H_2_ concentration reached 31.89 mol% with the lower Ni loading investigated. Although detailed tar yields and molecular compositions were not reported, the study supports the use of a separate hot catalytic stage to convert condensable hydrocarbons generated from heterogeneous SS–plastic-containing wastes [89].

The earlier study by Akkache et al. should be interpreted more cautiously. Plastic labels and wastewater sludge were gasified separately, and their individual properties were compared to assess their suitability for subsequent blending. Thus, the paper does not experimentally demonstrate a tar synergy for an actual SS–plastic mixture. Nevertheless, plastic labels showed approximately 87% conversion and generated a relatively energy-rich gas, leading the authors to identify them as a suitable co-feed for both secondary and digested wastewater sludge [172].

The main effect of increasing gasification temperature is clearly a reduction in total tar yield, accompanied by higher gas production. Plastic addition can assist this conversion because plastic-derived radicals interact with SS-derived oxygenated tar fragments and because sludge minerals may catalyze secondary reactions. However, the effect depends strongly on plastic type and loading. Moderate PP and PS fractions can enhance tar conversion, whereas excessive loading generates heavier hydrocarbons and waxes that may escape complete reforming. PE appears particularly favorable for gas production and H_2_ formation [86]. This point has practical importance because a low gravimetric tar concentration alone does not guarantee a low tar dew point or trouble-free syngas. Even small concentrations of heavy PAHs can be more problematic for downstream equipment than much larger concentrations of light aromatic compounds.

In terms of composition, SS initially contributes a substantial fraction of oxygenated tar, including phenolic, carbonyl and alcoholic species. PE and PP contribute predominantly aliphatic and olefinic intermediates, whereas aromatic-containing plastics such as PS have a greater intrinsic tendency toward aromatic products. With increasing secondary temperature, oxygenated and aliphatic compounds are preferentially cracked and reformed. Consequently, the absolute amount of tar decreases, but the remaining fraction may become enriched in thermally stable aromatics and PAHs. The staged experiments of Li et al. provide particularly clear evidence of this transition toward heavier multi-ring aromatics at 800 °C [171].

The literature also provides a strong rationale for integrating SS-derived char directly into SS–plastic co-gasification. SSC has already demonstrated high catalytic activity toward model tar conversion: Lu et al. achieved 97.1% toluene conversion at 950 °C in the presence of steam and CO_2_, with the mineral-rich carbon matrix contributing adsorption and catalytic sites [51]. Other work has shown that SSC can completely convert toluene and naphthalene initially above approximately 850 °C, although coke deposition causes progressive deactivation [173].

Overall, the available evidence suggests that SS–plastic co-gasification can provide genuine non-additive improvements in gas formation and secondary hydrocarbon conversion, but these effects are strongly dependent on polymer type, mixing ratio, and gasification severity. The clearest evidence is provided by Yang et al., where experimentally measured H_2_ and CH_4_ yields exceeded theoretically additive values, while heavier gaseous hydrocarbons were produced below the expected additive level [86]. This indicates enhanced secondary cracking rather than a simple dilution or heating-value effect. However, the interaction was not uniformly beneficial: PP and PS loadings above approximately 50 wt% promoted the formation of heavier hydrocarbons and waxes that were insufficiently converted, whereas PE remained more favorable for H_2_ production [86]. The staged experiments of Li et al. further demonstrate that the direction of the interaction can reverse with temperature, from increased condensable production at 700 °C to enhanced gas formation and tar conversion at 800 °C [171]. Thus, co-gasification synergy should be regarded as a condition-specific phenomenon rather than an intrinsic consequence of combining plastics with SS.

The role of SS is also inherently dual. Its oxygenated volatile products contribute substantially to tar formation, as demonstrated by the much higher tar concentration from SS than from the individual plastics under comparable conditions [86], but its moisture and mineral constituents may simultaneously facilitate steam reforming and catalytic cracking of plastic- and sludge-derived intermediates. Therefore, SS does not necessarily improve plastic gasification simply by being present; its benefit arises only when the resulting oxygenated intermediates can be effectively reformed and when the catalytic contribution of the sludge mineral phase outweighs additional tar formation. Evidence from more complex systems containing catalysts or simulated municipal wastes supports the value of secondary reforming, but does not isolate the contribution of SS–plastic interactions [89]. Likewise, the work of Akkache et al. should not be considered evidence of co-gasification synergy because the plastic and sludge feeds were gasified separately [172]. Consequently, the current literature supports SS–plastic co-gasification most strongly for H_2_-oriented and tar-reforming configurations operated at sufficiently high secondary temperatures, while direct comparative studies using plastic-only, SS-only, and theoretically additive reference cases remain limited. The effects of SS–plastic co-processing on gas production, tar formation, and tar composition, as reported in representative experimental studies, are summarized in Table 10.

### 4.5. Hydrothermal Carbonization

HTC treats plastics in hot compressed water, typically at 180–250 °C under autogenous pressure. Changes in water polarity, ionic product, and solvent properties promote hydrolysis, elimination, condensation, and aromatization, but polymer response depends strongly on chemical structure [174]. Plastics containing reactive or hydrolysable groups, such as PVC, PET, PC, and PA6, behave very differently from C–C backbone polymers such as PE, PP, and PS.

PVC is the most extensively studied example because HTC can simultaneously promote dechlorination and carbonization. Poerschmann et al. showed that chlorine removal became nearly complete above 235 °C, while solid carbon recovery decreased with increasing temperature [175]. Yao and Ma reported that increasing temperature from 200 to 260 °C reduced solid chlorine content from 42.7 to 3.03 wt.%, while increasing carbon content to about 82.5 wt.% and HHV to 36.87 MJ·kg^−1^ [176]. Similar improvements in carbon ordering and fuel quality were reported at 250 °C [177]. while ethanol–water mixtures increaseddechlorination efficiency to 96.33% [178].

Hydrolysable polymers follow different pathways. PC and PA6 can generate phenolic compounds, bisphenol A, and caprolactam, whereas PP remains comparatively resistant [179]. PET mainly undergoes ester hydrolysis and may form terephthalic acid rather than conventional hydrochar [180]. Near-complete conversion to monomeric products was reported at 250 °C [181].

Conventional PE, PP, and PS are comparatively resistant to water-only HTC. Their conversion generally requires acids, oxidants, microwave assistance, or subsequent high-temperature carbonization [182,183], and they may remain largely unchanged at 250 °C [181].

Overall, hydrothermal treatment of plastics should not be described by a single HTC mechanism. Depending on polymer type, it may involve dechlorination, hydrolysis, depolymerization, carbonization, or minimal chemical conversion. Distinguishing true HTC from hydrothermal depolymerization and assisted pretreatment is therefore essential when comparing reported products and mechanisms [181].

#### Hydrothermal Co-Carbonization of Plastics with SS

Hydrothermal co-carbonization (co-HTC) provides a potentially attractive route for the simultaneous valorization of SS and plastic wastes. SS is particularly suitable for hydrothermal processing because its inherent moisture can serve as the reaction medium, avoiding the energy-intensive drying required before conventional pyrolysis. However, sludge-derived hydrochar generally suffers from high ash and nitrogen contents, while plastics can introduce additional carbon and energy into the system. The available literature nevertheless remains remarkably limited: direct co-HTC studies have so far focused primarily on PVC and PET, while the behavior of PE, PP and PS in sludge has mainly been investigated in the context of microplastic degradation rather than their deliberate use as co-feedstocks.

The first systematic study of SS/PVC co-HTC was reported by Lu et al. (2021) [184]. SS and PVC were treated at 230 °C for 1 h with PVC proportions of 30–70wt%. The interaction between the two feedstocks was particularly important for both PVC dechlorination and heavy-metal behavior. Dehydrochlorination of PVC generated HCl in the aqueous phase, creating an acidic environment capable of promoting the dissolution and migration of metals from the solid. Consequently, concentrations of Zn and Mn in the co-HTC hydrochars were substantially lower than theoretically expected from independent carbonization of the two feedstocks, while relatively low PVC addition also reduced the concentrations and potentially bioavailable fractions of Ni and As. At the same time, sludge promoted PVC dechlorination, achieving efficiencies of approximately 69.5–81.4%. This synergistic interaction was attributed partly to hydroxyl-containing sludge components and metals such as Ni, which can facilitate PVC dechlorination, while the sludge matrix may also reduce PVC agglomeration and improve mass transfer. Among the studied compositions, an SS:PVC ratio of 7:3 provided a favorable compromise between contaminant removal and fuel quality, producing hydrochar with an HHV of 13.58 MJ kg^−1^ and improved combustion characteristics.

A complementary role of PVC was demonstrated by Xie et al. (2022) [185], who investigated SS/PVC co-HTC specifically for the production of low-nitrogen solid fuel. Nitrogen retention is a major limitation of SS HTC because proteins and other nitrogen-containing organic compounds can remain in the hydrochar and subsequently contribute to NO_x_ formation during combustion. PVC decomposition produced an acidic hydrothermal environment that promoted the hydrolysis of refractory sludge proteins into amino acids and facilitated the conversion of solid organic nitrogen into more readily removable inorganic and nitrile-N species. Under optimized conditions of 240 °C, 1.5 h and a water loading of 0.84 g cm^−3^, hydrochar containing only 0.94wt% N was obtained, corresponding to a denitrification efficiency of approximately 75%, although its HHV remained relatively modest at 9.84 MJ kg^−1^. Increasing water loading from 0.54 to 0.84 g cm^−3^ was particularly effective, decreasing hydrochar nitrogen from 1.50 to 0.94wt%. However, PVC addition exhibited a clear optimum: moderate addition promoted denitrification, whereas excessive PVC enhanced secondary polymerization and reincorporation of nitrogen-containing intermediates into the solid. High PVC loading also promoted the formation of more ordered, graphite-like carbon, which could reduce combustion reactivity despite increasing carbonization.

More recently, Ye et al. (2024) [186] extended the concept from PVC to PET. In this case, PET acts differently because its ester bonds undergo hydrolysis under hydrothermal conditions, producing terephthalic acid and ethylene glycol. The formation of terephthalic acid provides an in situ acidic environment that can promote sludge-protein hydrolysis, while PET simultaneously acts as an additional carbon source. However, the stable extracellular polymeric substances (EPS) surrounding sludge cells can restrict the release of intracellular and proteinaceous nitrogen. Ye et al. therefore pretreated the sludge using alkali, ultrasonic cell disruption and sodium dodecyl sulfate, individually and in combination. Combined SDS–alkali pretreatment effectively loosened the EPS structure and facilitated protein release before co-HTC. Under optimized conditions of 240 °C for 2 h, with 20wt% PET and a water loading of 0.6 g cm^−3^, a hydrochar containing only 0.51wt% N was obtained. The study therefore demonstrates a complementary synergy: sludge pretreatment makes protein nitrogen accessible, while PET hydrolysis creates the acidic environment required to promote its subsequent conversion and removal.

Studies on microplastics in SS provide additional mechanistic evidence for these interactions. Xu and Bai (2022) reported a 79% reduction in naturally occurring microplastics during sludge HTC at 260 °C and demonstrated that PET was much more susceptible to degradation than PP because its ester bonds readily undergo hydrolysis [187]. Similarly, Li et al. (2022) [188] showed that PE, PS and PET underwent greater surface degradation and leaching in SS than in pure water, with sludge proteins and carbohydrates promoting polymer aging and depolymerization. PET again exhibited the strongest transformation. More recent work has shown that even relatively resistant PE can undergo partial degradation during prolonged HTC in a sludge matrix, with approximately 71% removal reported at 220 °C after 3 h [95]. These results suggest that the organic and inorganic components of sludge can actively alter plastic transformation rather than merely acting as an inert co-feedstock.

Overall, the available evidence indicates that the advantages of SS–plastic co-HTC arise primarily from polymer-specific chemical interactions rather than from simple co-processing of two waste streams. The strongest evidence is available for PVC and PET, whose hydrothermal decomposition generates acidic species that actively modify sludge conversion. In SS/PVC systems, PVC-derived HCl promotes protein hydrolysis, nitrogen removal and heavy-metal redistribution, whereas sludge components simultaneously enhance PVC dechlorination [184,185]. PET provides a related but chemically distinct effect through hydrolysis to terephthalic acid, which can acidify the reaction medium and promote nitrogen removal from pretreated sludge [186]. These results demonstrate genuine functional complementarity, but also show that the optimum plastic fraction must be carefully controlled because excessive PVC can promote secondary polymerization, nitrogen reincorporation and formation of more ordered carbon structures with lower combustion reactivity [185].

At the same time, the current evidence base remains too limited to establish co-HTC as a broadly advantageous strategy for mixed plastic wastes. Most direct co-feed studies concern PVC and PET, while the reported transformation of PE, PP and PS is derived mainly from studies of microplastics already present in sludge rather than deliberately prepared SS–plastic mixtures [95,187,188]. Such observations provide useful mechanistic evidence that the sludge matrix can accelerate polymer aging or degradation, but they do not demonstrate improved process performance relative to plastic-only or SS-only HTC. Moreover, enhanced metal mobilization under acidic conditions may represent either an advantage for subsequent recovery or an environmental liability depending on downstream liquid-phase management [184]. Consequently, the principal benefit of co-HTC currently appears to be the exploitation of chemically reactive polymers to assist sludge dechlorination, denitrification and contaminant redistribution, rather than a universal enhancement of plastic conversion. Systematic studies incorporating single-feed controls, theoretically additive reference cases, and complete balances of carbon, nitrogen, chlorine, metals and phosphorus are still required before the observed PVC- and PET-specific interactions can be generalized to practical mixed-plastic/SS systems.

### 4.6. Hydrothermal Liquefaction

HTL converts waste plastics in subcritical, near-critical, or supercritical water into liquid hydrocarbons or recoverable monomers. Near the critical point of water (374 °C, 22.1 MPa), its lower dielectric constant improves the solubility of non-polar organics and promotes ionic and free-radical reactions. Polymer structure is therefore decisive: polyolefins require high-temperature radical cracking, whereas polymers with hydrolysable groups can decompose at lower temperatures [189,190].

PE shows limited conversion under subcritical conditions because of its strong C–C/C–H backbone. More than 90% residual solids were reported after treatment at 350 °C for 20 min [189], while efficient conversion generally requires >400–425 °C. Oil yields of about 80–90% have been reported at 425–530 °C, producing mainly alkanes, alkenes, and increasingly cyclic/aromatic hydrocarbons [191,192,193,194,195]. PP behaves similarly, although reported oil yields vary widely with reactor configuration and recovery method: values range from 32% at 425 °C to about 91% at 450 °C [190,196]. PP-derived oils can reach HHVs around 46.3 MJ·kg^−1^ [194].

PS is more readily converted because benzylic bond cleavage is favorable. Oil yields of 70.6–93% have been reported between 350 and 450 °C, with styrene and aromatic dimers as primary products and increasing formation of toluene, ethylbenzene, cumene, and polyaromatics at higher severity [190,194]. PET mainly undergoes hydrolytic depolymerization to terephthalic acid and ethylene glycol rather than hydrocarbon oil formation; reported oil yields are therefore low, and monomer recovery is a more suitable target [189,190]. PVC undergoes hydrothermal dechlorination, releasing HCl and favoring char formation; although chlorine removal can be substantial, PVC complicates mixed-plastic HTL because of acidification and corrosion [189].

Overall, HTL is most promising for producing hydrocarbon-rich oils from PE, PP and PS under near- or supercritical conditions, whereas PET is better suited to monomer recovery and PVC to hydrothermal dechlorination. This polymer-specific behavior should therefore be considered when designing HTL processes for realistic mixed-plastic wastes.

#### Co-Hydrothermal Liquefaction of Plastics and SS

Research specifically addressing the simultaneous HTL of plastics and SS remains very limited. Existing studies have mainly examined the behavior of microplastics already embedded in municipal SS, rather than deliberately blending bulk waste plastics with sludge at controlled ratios. Nevertheless, the available work indicates that plastics can significantly influence product phase behavior and biocrude quality during sludge HTL.

An early indication of this interaction was reported by Prestigiacomo et al. (2019) [197] during catalytic HTL of real SS at 325 °C for 30 min. Although the primary objective was to compare SS with *Chlorella vulgaris* as an HTL feedstock, the authors observed the formation of an unusual additional organic phase during catalytic treatment of SS that did not appear during HTL of microalgae. The phase accounted for approximately 5–10wt% of the dry feed and was tentatively attributed to microplastics accumulated in the sludge. FTIR analysis revealed bands associated with C–Cl bonds, suggesting the contribution of PVC-containing microplastics. The study therefore provided the first evidence that synthetic polymers naturally present in SS can follow conversion pathways distinct from those of proteins, lipids and carbohydrates and can form a separate wax-like fraction during HTL.

This phenomenon was investigated more systematically by Prestigiacomo et al. (2022) [198] using real municipal sludge containing approximately 3 ± 1wt% microplastics. HTL was again performed at 325 °C for 30 min, both without catalyst and using CoMo/Al_2_O_3_, NiMo/Al_2_O_3_ and activated carbon felt. A distinct additional phase was generated only in the catalytic experiments, with yields of approximately 5, 8 and 6wt% on a dry ash-free basis for CoMo/Al_2_O_3_, NiMo/Al_2_O_3_ and activated carbon felt, respectively. This fraction differed chemically and physically from conventional biocrude and contained oleamide as well as chlorinated organic compounds. Controlled hydrothermal experiments with model plastics further supported the conclusion that the additional wax-like phase originated from synthetic polymers present in the sludge.

The results also demonstrate that plastic contamination may adversely affect HTL product quality. Chlorinated compounds originating from PVC can transfer into intermediate organic fractions, increasing the heteroatom content and potentially raising the severity and cost of downstream upgrading. Hydrothermal treatment of PVC at 325 °C generated an acidic aqueous phase with a pH of approximately 2.6 and left about half of the initial PVC as solid residue, confirming that dechlorination and incomplete polymer conversion occur simultaneously under these relatively mild HTL conditions. Consequently, the plastic fraction in SS cannot simply be considered an inert contaminant. Its composition may affect corrosion, phase separation and the quality of the resulting biocrude [198].

More broadly, recent work by Silva Thomsen et al. (2024) [199] demonstrates the considerable potential of SS HTL itself, with maximum biocrude yields generally obtained around 325 °C and positive synergistic interactions observed when different wastewater-treatment residues were processed together. Although plastics were not deliberately co-fed in that study, the authors explicitly identify microplastic destruction as an additional environmental benefit of sludge HTL. Their results also show that sludge composition strongly controls product distribution: protein-rich secondary sludge tends to produce more aqueous-phase products, whereas lipid-rich fractions favor biocrude formation. This compositional sensitivity suggests that deliberately introduced plastics could further alter carbon partitioning and reaction pathways, particularly if reactive intermediates from plastic decomposition interact with sludge-derived lipids, proteins or carbohydrates.

Overall, the currently available evidence is insufficient to conclude that deliberate SS–plastic co-HTL provides a general synergistic advantage over the separate liquefaction of either feedstock. The studies of Prestigiacomo et al. demonstrate that microplastics naturally present in municipal sludge can substantially alter phase separation and product composition during HTL, but these systems differ fundamentally from controlled co-processing experiments with defined plastic type and loading [197,198]. In particular, the formation of an additional wax-like organic phase under catalytic conditions shows that synthetic polymers are not inert during sludge HTL; however, the presence of chlorinated compounds and incomplete PVC conversion also indicates that plastic-derived products may deteriorate biocrude quality and increase downstream upgrading and corrosion requirements [197,198]. Thus, the observed interaction should presently be interpreted as evidence of process modification, rather than as proof of beneficial co-liquefaction synergy.

The role of SS in such systems also remains unresolved. Unlike co-HTC, where acidic products generated from PVC or PET can directly promote sludge denitrification or contaminant redistribution, the available co-HTL studies do not yet demonstrate that SS improves the conversion of plastics themselves. Moreover, the strong dependence of SS HTL performance on sludge biochemical composition suggests that any future interaction with plastics is likely to be highly feedstock-specific [199]. Consequently, the main scientific question is not whether plastics influence sludge HTL—this has already been demonstrated—but whether controlled addition of specific polymers can improve biocrude yield, quality, carbon recovery, or heteroatom removal relative to appropriate single-feed and theoretically additive reference cases. Until such experiments are available, co-HTL should be considered a promising but insufficiently validated integration pathway, with current evidence highlighting both potential opportunities for microplastic destruction and important risks associated with phase separation and chlorine-containing products [197,198,199].

## 5. Deactivation, Regeneration, and Reuse of SSA- and SSC-Based Catalysts

Catalyst stability is an important consideration for the practical application of SSA- and SSC-based materials during pyrolysis, gasification, and secondary reforming of plastics. Unlike relatively homogeneous commercial catalysts, sludge-derived materials contain variable mixtures of Fe-, Ca-, Al-, Si-, P-, S-, alkali-, and trace-metal-containing phases, while SSC additionally contains a porous carbonaceous matrix. Consequently, deactivation may involve several simultaneous mechanisms, particularly coke formation, pore blockage, mineral sintering, and chemical transformation of active phases by chlorine and sulfur. Direct investigations of catalyst deactivation during plastic conversion remain scarce, particularly for SSA. Nevertheless, studies of SSC during plastic pyrolysis and tar cracking provide useful mechanistic evidence.

Coke formation is probably the most important deactivation pathway for SSC. Plastic-derived olefins, aromatics, and heavier intermediates can undergo secondary condensation, dehydrogenation, and polymerization on catalytic surfaces, generating progressively less reactive carbonaceous deposits. Because SSC activity depends on both its porous carbon matrix and accessible inorganic phases, coke can simultaneously cover Fe-, Ca-, Al-, and other active sites and restrict transport through the pore network. Lu et al. demonstrated this directly during catalytic cracking of toluene over SSC: coke deposition decreased the BET surface area from 74.2 to 51.8 m^2^ g^−1^ under N_2_ [51]. Importantly, CO_2_ and steam reduced the decline in surface area by promoting gasification of deposited carbon. Similarly, Fuentes-Cano et al. (2013) attributed time-dependent deactivation of sewage-sludge-derived char during toluene and naphthalene conversion to soot deposition on active sites [173]. Increasing temperature and steam concentration enhanced soot gasification and therefore reduced deactivation.

These observations are directly relevant to plastic pyrolysis because olefin-rich vapors from PE and PP and aromatic intermediates from PS provide potential coke precursors. More importantly, Sun et al. demonstrated that SSC can catalytically promote secondary conversion and aromatization of mixed PE/PP/PS pyrolysis vapors [20]. Changes in catalytic performance after use indicated progressive modification of the active surface, emphasizing that coke accumulation and transformations of the sludge-derived mineral phases need to be considered when SSC is repeatedly applied.

Ash deposition, pore blockage, and sintering should be distinguished between SSA and SSC. SSC contains an ash-rich mineral fraction embedded within carbonaceous material. Therefore, deposited coke and condensed or partially molten inorganic species can progressively reduce accessible porosity. SSA, in contrast, contains essentially no combustible carbon matrix and usually exhibits a relatively low specific surface area, meaning that its activity is primarily associated with exposed mineral surfaces. For pre-produced SSA employed with relatively clean PE, PP, PET, or PS, external ash deposition should be less important than during direct SS–plastic co-processing. Instead, repeated thermal exposure can promote sintering, agglomeration, and phase transformations of Ca-, Fe-, Al-, Si-, and P-containing species, decreasing dispersion and accessibility of active sites. This mechanism is plausible for SSA but has not yet been systematically quantified during repeated plastic-pyrolysis cycles.

Chlorine poisoning and pore blockage are particularly important when PVC is present. PVC undergoes extensive dehydrochlorination during pyrolysis, producing HCl, and SS–plastic co-pyrolysis experiments confirm substantially enhanced chlorine release when PVC is incorporated [84]. Lu et al. showed that HCl reacted with inorganic components of SSC to generate low-melting chlorides. Above approximately 750 °C, these compounds could melt and obstruct pores. Addition of only 300 ppm HCl decreased toluene conversion at 850 °C from 86.0 to 81.2% [51]. Chlorination may therefore deactivate both SSC and SSA by transforming catalytically active Ca-, Fe-, or alkali-containing phases and promoting their redistribution or agglomeration. PVC can also alter heavy-metal partitioning during SS–plastic co-pyrolysis [84].

The influence of sulfur and heavy metals is more complex than conventional catalyst-poisoning terminology suggests. Sulfur should not automatically be regarded as detrimental to unmodified SSA or SSC because sulfides and sulfur-associated metal phases may themselves participate in catalytic reactions. Sulfur poisoning is expected to become particularly important for metal-loaded SSC catalysts, especially Ni-containing materials, because H_2_S and other sulfur compounds can convert metallic Ni into less active sulfide phases. Likewise, sewage-sludge-derived solids commonly retain and concentrate metals such as Cu, Zn, Cr, Ni and Pb during thermal treatment [200,201]. Depending on their chemical form, these metals can act as catalytic components, spectators, or contaminants. Therefore, high total heavy-metal concentration alone does not demonstrate poisoning, and changes in oxidation state, mineral association, dispersion, volatility, and accessibility are more relevant indicators of deactivation.

Regeneration and reuse remain major challenges, particularly for SSC. Conventional oxidative regeneration is potentially suitable for SSA because deposited carbon can theoretically be combusted while retaining the non-combustible mineral catalyst. However, excessive regeneration temperatures can promote irreversible sintering and mineral-phase transformations. For SSC, regeneration is more complicated because both the unwanted coke and the catalyst support are carbonaceous. Aggressive oxidation can therefore remove the SSC matrix together with deposited coke.

Regeneration using steam or CO_2_ is potentially more suitable because carbon deposits can be gasified under controlled conditions. The beneficial effects of steam and CO_2_ on maintaining char activity have been demonstrated during tar conversion [51,173], although excessive gasification would ultimately consume the SSC itself. Metal modification can further improve stability. Daorattanachai et al. showed that addition of Re to Ni/SSC improved Ni dispersion and resistance to carbon deposition, while steam and limited O_2_ further reduced coke formation [161], and only an approximately 5% deactivation in H_2_ yield occurred after 18 h under optimized conditions. Fe/Ni supported on municipal sludge char has likewise shown comparatively stable performance during extended tar reforming [200]. More recently, activated Ni/Ca-functionalized sludge-derived biochar maintained relatively stable H_2_ production over five reuse cycles [160].

Overall, SSA is likely to be more compatible with oxidative coke regeneration, whereas SSC may be better regarded as a low-cost, partially regenerable or ultimately sacrificial catalyst. However, this distinction requires direct experimental confirmation during plastic conversion. Future studies should therefore combine multi-cycle catalytic tests with BET analysis, temperature-programmed oxidation of coke, SEM-EDX, XRD, XPS, and elemental analysis to distinguish reversible carbon deposition from irreversible sintering, chlorination, sulfidation, metal redistribution, and mineral-phase transformation.

## 6. Techno-Economic Analysis and Life-Cycle Assessment

Quantitative techno-economic analysis (TEA) and life-cycle assessment (LCA) specifically addressing integrated sewage sludge–plastic thermochemical co-valorization remain limited. Consequently, available assessments of thermochemical plastic recycling, sewage-sludge conversion, and related waste co-processing systems should primarily be regarded as quantitative benchmarks rather than as directly transferable estimates for SS–plastic systems. Nevertheless, these studies identify the principal economic and environmental parameters that are likely to determine the feasibility of integrated processing, including feedstock cost and moisture content, plant capacity, product yield and value, energy requirements, transportation, avoided waste-management burdens, and credits associated with energy or material recovery.

Direct quantitative environmental evidence is already available for some SS–plastic co-processing systems. Jadlovec et al. investigated a 1:1 sewage sludge–polyethylene blend in a fluidized-bed combustor and complemented the experimental study with a gate-to-gate LCA. The lower heating value of the blend reached 16.29 MJ kg^−1^, compared with 9.83 MJ kg^−1^ for sewage sludge and 31.81 MJ kg^−1^ for polyethylene. Using a functional unit corresponding to 1 h of stable incineration, the climate-change impact was 1.6167 kg CO_2_-eq for the SS-PE mixture, compared with 1.8084 kg CO_2_-eq for PE and 1.8645 kg CO_2_-eq for SS. The mixed fuel showed lower impacts in eight of the nine evaluated impact categories, although NO_x_ and SO_2_ emissions remained substantially higher than for pure PE [93].

A related techno-economic benchmark was provided by Alves et al., who evaluated a small-scale gasification plant processing sewage sludge together with solid recovered fuel. At a processing capacity of 883 kg h^−1^, the electricity-production scenario resulted in a payback period of 9 years and an internal rate of return (IRR) of 7.5%. The hydrogen-production scenario showed a longer payback period of 12 years and an IRR of 4.3%, but achieved a higher end-of-life net present value of approximately EUR 1.80 million. Sensitivity analysis indicated that capital expenditure was one of the most influential parameters affecting payback period and IRR. Although solid recovered fuel is not equivalent to a defined plastic feedstock, this study provides a useful quantitative benchmark for thermochemical systems combining sewage sludge with a combustible waste-derived co-feedstock [202].

For mixed-plastic thermochemical recycling, Yadav et al. evaluated a conceptual catalytic fast-pyrolysis facility processing 240 metric tons day^−1^ of mixed plastic waste. The modeled base case produced a minimum selling price (MSP) of USD 1.07 kg^−1^ for a benzene–toluene–xylenes (BTX) product stream, compared with approximately USD 0.68 kg^−1^ for virgin BTX. Economic competitiveness was predicted when the mixed-plastic feedstock cost decreased below approximately USD 0.10 kg^−1^ or crude-oil prices exceeded USD 60 barrel^−1^. From an environmental perspective, catalytic fast pyrolysis reduced total supply-chain energy use by approximately 24%; however, GHG emissions per kilogram of BTX were approximately 2.4 times those of conventional fossil-based BTX production. These findings demonstrate that lower fossil-energy consumption does not necessarily correspond to lower life-cycle GHG emissions [15].

Similar economic–environmental trade-offs have been reported for mixed-plastic gasification by Afzal et al., with estimated MSP values of USD 0.70 kg^−1^ for methanol and USD 3.41 kg^−1^ for hydrogen produced from mixed plastic waste. The economics were particularly sensitive to syngas yield and plastic-feedstock cost, with feedstock prices below approximately USD 0.02 kg^−1^ allowing the modeled methanol pathway to approach cost parity with conventional production. Relative to fossil-based routes, total supply-chain energy requirements decreased by approximately 52% for methanol and 56% for hydrogen, whereas GHG emissions increased by approximately 166% and 36%, respectively. The cradle-to-gate LCA nevertheless showed lower impacts than the corresponding fossil pathways in several categories, including smog formation, acidification, non-carcinogenic human-health impacts, ozone depletion, eutrophication, and particulate formation, primarily because of credits assigned to exported steam and electricity [16].

More recent modeling further illustrates the strong dependence of plastic-pyrolysis economics on operating assumptions, feedstock cost, and plant scale. Zaker Hosseiny and Shi estimated a minimum pyrolysis-oil selling price of approximately USD 420 t^−1^ under the most favorable modeled conditions for a baseline plant capacity of 100 kt y^−1^. However, Monte Carlo analysis accounting for technical and economic uncertainty yielded a mean MSP of approximately USD 1137 t^−1^. The modeled process remained competitive with the selected crude-oil benchmark only when waste-polypropylene feedstock cost remained below approximately USD 320 t^−1^. Increasing plant capacity beyond approximately 90 kt y^−1^ provided only limited additional economies of scale, while capital expenditure and transportation costs were identified as important economic drivers. These findings emphasize that apparently favorable deterministic TEA results can change substantially when feedstock variability and process uncertainty are incorporated into the analysis [203].

Quantitative studies of sewage-sludge conversion show similarly strong dependence on the selected technology and regional assumptions. In an Indian case study based on process simulation, Vidyarthi et al. compared anaerobic digestion, gasification, pyrolysis, and hydrothermal liquefaction of sewage sludge. The estimated energy outputs were approximately 0.76 kWh kg^−1^ of dry sludge for gasification, 0.53 kWh kg^−1^ for anaerobic digestion, 0.34 kWh kg^−1^ for pyrolysis, and 0.13 kWh kg^−1^ for HTL. The corresponding levelized costs of electricity were approximately USD 0.11 kWh^−1^ for gasification, USD 0.02 kWh^−1^ for anaerobic digestion, USD 0.14 kWh^−1^ for pyrolysis, and USD 2.21 kWh^−1^ for HTL. These values demonstrate that maximizing energy recovery alone does not necessarily maximize economic performance and that conclusions depend strongly on technology configuration and local economic assumptions [204].

The high moisture content of sewage sludge is particularly important when defining system boundaries because conventional pyrolysis and gasification generally require dewatering and drying, whereas hydrothermal technologies can process wet feedstocks directly. Ranganathan evaluated renewable-fuel production from sewage sludge through HTL and estimated an MSP of approximately USD 4.04 per gasoline-gallon equivalent. Sensitivity analysis identified biocrude yield and operating cost as major economic drivers, emphasizing the importance of conversion efficiency and process-energy requirements in determining the economic viability of sludge-derived fuels [205].

More recently, Kapusta et al. evaluated continuous HTL of municipal sewage sludge followed by biocrude upgrading using experimentally based process data. The modeled system resulted in a dynamic generation cost of approximately USD 0.90 L^−1^, an NPV of USD 2.93 million, and an IRR of 5.2%. The upgraded fuel had a reported carbon footprint of approximately 19 g CO_2_-eq MJ^−1^, corresponding to approximately 80% GHG savings relative to the fossil-diesel benchmark adopted in the study. This example demonstrates the potential environmental advantage of processing wet sludge without extensive pre-drying, although the resulting economics remain sensitive to scale, upgrading requirements, and product value [206].

LCA studies of sewage-sludge pyrolysis further demonstrate the strong influence of product utilization and process-energy integration. Huang et al. compared sewage-sludge pyrolysis routes oriented toward bio-oil, biochar, and activated-carbon production using a cradle-to-gate system boundary and a functional unit of 1000 kg of dried sewage sludge entering the pyrolysis process. The reported climate-change impacts for the bio-oil- and biochar-oriented scenarios corresponded to approximately −0.31 and −0.05 kg CO_2_-eq per kilogram of functional-unit sludge, respectively. Internal utilization of pyrolysis gas reduced external process-energy demand by approximately 21–34%, although the associated effect on global warming potential depended on the particular scenario. Product yield and market value were also identified as major determinants of economic performance, while the authors noted that more complete capital-cost assessment would be required for comprehensive economic evaluation [207].

The environmental and economic performance of sewage-sludge pyrolysis also depends strongly on which conventional sludge-management route is assumed to be displaced. Faragò et al. combined LCA and cost–benefit analysis to compare sewage-sludge pyrolysis with agricultural application and incineration. Pyrolysis reduced impacts in several environmental categories and produced a positive NPV relative to agricultural application. However, biochar-oriented pyrolysis was less economically favorable than incineration under the evaluated conditions, with NPVs approximately EUR 19–22 t^−1^ of sludge lower than those of the incineration reference. The comparison was particularly sensitive to the assumed CO_2_ externality price of EUR 164 t^−1^ CO_2_-eq and, for activated-carbon scenarios, to the assumed pharmaceutical-micropollutant removal performance of sludge-derived activated carbon. These results emphasize that both environmental credits and economic conclusions depend strongly on the selected system boundaries and substitution assumptions [208].

Hosseinian et al. similarly demonstrated that the environmental attractiveness of sewage-sludge pyrolysis depends on the existing sludge-treatment route and the credits assigned to recovered materials. In case studies of two Swedish municipalities, avoided production of mineral fertilizers contributed substantially to the environmental benefits of several sludge-management configurations. Combining anaerobic digestion with subsequent pyrolysis provided particularly favorable environmental performance in the evaluated scenarios while also reducing the risk of transferring contaminants to agricultural soil. These findings are relevant to SS–plastic co-processing because the fate and subsequent utilization of the inorganic fraction of sewage sludge can substantially affect the overall environmental balance of an integrated thermochemical system [209].

Additional evidence from SS co-pyrolysis with non-plastic co-feedstocks illustrates how process integration itself can influence life-cycle performance. A comparative LCA of sewage-sludge co-pyrolysis with agricultural and forest residues reported reductions in individual environmental-impact indicators of approximately 10–90%, while overall environmental impacts decreased by up to approximately 48%. The best-performing scenario achieved a maximum net positive energy balance of +1368 kWh per functional unit. Sensitivity analysis identified bio-oil yield and feedstock transportation among the most influential parameters. Although lignocellulosic residues behave differently from plastics during pyrolysis, the study provides an important methodological benchmark by showing that the environmental benefits of co-processing can be highly sensitive to co-feedstock logistics, product distribution, and substitution credits [210].

Taken together, these studies demonstrate that the economic and environmental performance of SS–plastic co-processing cannot be inferred solely from increased conversion, liquid yield, syngas production, or heating value. A meaningful TEA/LCA of an integrated SS–plastic system should include plastic collection, sorting, and pretreatment; sludge thickening, dewatering, and, where required, drying; transportation of both feedstocks; reactor heating and auxiliary electricity; catalyst preparation, activation, replacement, and regeneration; gas and liquid upgrading; treatment of aqueous streams; ash and contaminant management; and final utilization of the resulting products.

For systems involving SS-derived catalysts, additional burdens associated with acid or alkaline treatment, washing, solvent consumption, metal impregnation, thermal activation or calcination, and catalyst-to-plastic ratio should also be considered rather than treating the waste-derived catalyst as an environmentally burden-free material. This is particularly important for highly modified SS-derived catalysts because improvements in catalytic activity may be accompanied by substantial chemical consumption, energy demand, wastewater generation, and additional processing steps.

At the same time, integrated SS–plastic processing may generate environmental and economic credits that are absent from conventional single-feedstock assessments. These may include avoided sewage-sludge disposal or incineration burdens, reduced consumption of commercial catalysts, internal utilization of non-condensable gases for process heat, phosphorus or other mineral-resource recovery, displacement of fossil fuels or virgin petrochemicals, and potential utilization of residual ash or char. The magnitude of these credits is expected to depend strongly on regional waste-management practices, electricity and heat supply, transport distance, product quality, and the actual substitution pathway assumed for each recovered material.

The available literature indicates that both thermochemical plastic recycling and sewage-sludge valorization can be technically feasible while exhibiting substantial economic and environmental trade-offs. Direct quantitative assessments of systems combining sewage sludge and plastics are beginning to emerge, particularly for co-incineration, but comprehensive TEA/LCA studies of bulk SS–plastic co-pyrolysis, co-gasification, hydrothermal co-processing, and SS-derived-catalyst-assisted plastic conversion remain comparatively scarce. Consequently, future studies should integrate experimentally validated mass and energy balances with process simulation, capital and operating cost estimation, and harmonized cradle-to-gate or cradle-to-grave LCA. Sensitivity and uncertainty analyses should explicitly address feedstock moisture, sludge and plastic availability, transportation distance, plant scale, catalyst loading and lifetime, product yields, energy source, pollutant-control requirements, and avoided waste-management and product-substitution credits. Such assessments are required before the technical synergies reported for SS–plastic co-processing can be translated into robust conclusions regarding its economic and environmental advantages.

## 7. Problems and Perspectives

Despite the growing interest in SS-derived catalysts for pyrolysis, several important problems still limit their practical application. One of the main challenges is the strong variability of SS composition. The amount of organic matter, ash, minerals, heavy metals, phosphorus, sulfur, and nitrogen-containing compounds depends on the wastewater source, treatment technology, season, and sludge stabilization method. As a result, the catalytic properties of sludge-derived char or ash can differ significantly from one sample to another. This makes it difficult to compare results between studies and to develop universal catalyst preparation methods.

Another key limitation is the insufficient control over catalyst structure and active sites. SS-derived catalysts often contain complex mixtures of Si, Al, Ca, Fe, Mg, P, and other inorganic components. These species can promote cracking, reforming, deoxygenation, dehydrogenation, and aromatization reactions, but their individual roles are not always clearly identified. In many studies, the catalytic effect is described only through product yields, while detailed characterization of active phases, acidity/basicity, pore structure, and metal dispersion is limited. Therefore, deeper investigation of structure–activity relationships is necessary.

Catalyst deactivation is also a serious issue. During pyrolysis, sludge-derived catalysts can be deactivated by coke deposition, pore blockage, sintering of mineral particles, and contamination with chlorine, sulfur, or heavy metals from the feedstock. This is especially important when processing plastic waste, tires, textiles, or mixed waste, since these materials may contain additives, dyes, fillers, and halogenated compounds. However, regeneration and reuse of SS-derived catalysts are still poorly studied. Future research should focus not only on catalytic activity, but also on catalyst lifetime, regeneration efficiency, and stability after several reaction cycles.

From a technological point of view, most studies are still performed at the laboratory scale using model feedstocks and small reactors. Scale-up remains challenging because real waste streams are heterogeneous and pyrolysis product distribution is highly sensitive to temperature, heating rate, residence time, catalyst-to-feedstock ratio, and reactor configuration. More pilot-scale experiments are needed to evaluate the real performance of sludge-derived catalysts under continuous operation.

Nevertheless, SS-derived catalysts have strong potential for future development. They combine waste valorization, resource recovery, and catalytic upgrading in one approach. Instead of considering SS only as a disposal problem, it can be converted into low-cost functional materials for thermochemical conversion processes. Future perspectives include the controlled activation of sludge char, modification of sludge ash, metal loading, preparation of composite catalysts, and optimization of catalyst properties for specific feedstocks such as biomass, plastics, tires, textiles, and mixed municipal waste.

Overall, future research should move from simple yield comparison toward a more systematic evaluation of catalytic mechanisms, product quality, catalyst stability, environmental safety, and techno-economic feasibility. Such studies will help determine whether SS-derived catalysts can become a practical and sustainable alternative to conventional commercial catalysts in waste pyrolysis.

## 8. Conclusions

The thermochemical co-conversion of plastic waste and sewage sludge (SS) offers a promising strategy for integrating the management of two heterogeneous waste streams while recovering energy, chemicals, and materials. Their complementary properties provide the basis for this integration. Plastics generally have high volatile-matter, carbon, and hydrogen contents, low ash contents, and high heating values, whereas SS contains substantial moisture, oxygenated and nitrogen-containing organic matter, and a mineral-rich fraction containing Si, Al, Fe, Ca, Mg, P, and other elements. Consequently, SS–plastic conversion involves not only simultaneous processing but also interactions between decomposition intermediates and catalytic effects associated with sludge-derived minerals, which can modify reaction pathways and product distributions.

The importance of these interactions depends on the thermochemical route. Co-incineration mainly exploits energetic complementarity, because the high heating value of plastics can improve ignition, combustion intensity, and energy recovery from SS and may reduce supplementary-fuel requirements. However, these advantages must be balanced against NO_x_ and N_2_O formation from sludge-bound nitrogen, SO_2_ emissions, PVC-derived HCl, slagging, heavy-metal volatilization, and possible chlorinated pollutant formation. Co-incineration is therefore primarily an energy-recovery pathway rather than a chemical-recycling route.

Among the reviewed technologies, co-pyrolysis provides the clearest evidence of non-additive interactions. Hydrogen-rich radicals generated from PE and PP can interact with oxygenated and nitrogen-containing SS-derived intermediates, promoting hydrogen transfer, volatile formation, cracking, and changes in liquid composition. PS behaves differently because its aromatic rings are already present in the polymer structure, so SS mainly alters the distribution among styrene, monoaromatics, oligomers, and PAHs. PET produces oxygenated aromatic intermediates and therefore benefits mainly from decarboxylation, decarbonylation, and deoxygenation. PVC remains the most challenging polymer because dehydrochlorination precedes cracking and aromatization, while released chlorine can cause corrosion, catalyst deactivation, and increased metal volatility. Thus, no universal synergistic effect or optimum SS/plastic ratio can be applied across different polymers.

A key outcome of this review is the mechanistic distinction between SSA and SSC as catalytic materials. SSA acts predominantly through its mineral phase: Al- and Si-containing sites can contribute acidity and cracking activity, Fe-containing phases provide redox and dehydrogenation functionality, and Ca- and Mg-containing phases can participate in basic reactions, hydrogen transfer, and partial HCl capture in PVC-containing systems. SSC retains these mineral phases within a porous carbonaceous matrix that additionally provides adsorption and vapor-concentration sites and can facilitate radical stabilization, hydrogen transfer, surface dehydrogenation, and secondary condensation. SSC therefore behaves as a bifunctional material in which carbon-mediated and mineral-catalyzed reactions occur simultaneously. Activation, acid or alkaline treatment, zeolitization, and loading with Ni, Fe, Co, Ca, or bimetallic species can further improve catalytic activity, although performance cannot be predicted from bulk elemental composition alone.

Co-gasification is another promising integration route, particularly when H_2_-rich syngas and tar conversion are targeted. Available studies indicate that plastic addition can increase H_2_, CH_4_, and light-hydrocarbon formation while enhancing the conversion of SS-derived oxygenated tars. PE appears particularly favorable for H_2_ production, whereas excessive PP or PS addition can increase heavier hydrocarbons and waxes that are more difficult to reform completely. Combining SS–plastic co-gasification with an in situ or downstream bed of SSC, SSA, or metal-modified SS-derived catalyst therefore represents an attractive but insufficiently investigated configuration.

Hydrothermal processing offers an additional advantage because wet SS can be treated without complete drying. However, polymer behavior is highly specific. PVC can create an in situ acidic environment through dehydrochlorination, affecting SS denitrification and metal mobility, whereas PET hydrolysis generates terephthalic-acid-derived species that modify sludge conversion. PE, PP, and PS are much more resistant under conventional HTC conditions. Deliberate co-HTC and especially co-HTL of bulk plastics with SS remain poorly investigated, while most HTL evidence derives from microplastics already present in municipal sludge. Phosphorus transformation and recovery during SS–plastic co-HTC also remain largely unexplored.

Practical application of SS-derived catalysts is constrained by coke deposition, pore blockage, mineral sintering, ash accumulation, and poisoning by sulfur, chlorine, and heavy metals. SSC regeneration is particularly challenging because oxidative coke removal can simultaneously consume the carbonaceous catalyst matrix. In some applications, once-through use followed by energy or mineral recovery may therefore be more realistic than repeated regeneration.

Overall, the greatest opportunity lies in moving beyond simple blending toward an integrated thermochemical platform in which SS performs three complementary functions: as a co-feedstock, as a source of reactive intermediates, and as a precursor of catalytic materials. Distinguishing genuine feedstock synergy from mineral catalysis and heat- and mass-transfer effects remains a central challenge. Future studies should combine mechanistic experiments with comprehensive catalyst characterization, elemental and contaminant balances, long-term stability testing, continuous and pilot-scale operation, techno-economic analysis, and life-cycle assessment. Such integration could combine plastic recycling, SS valorization, product upgrading, energy recovery, and potentially phosphorus and metal recovery within a more comprehensive circular waste-management strategy, while reducing reliance on virgin resources and conventional catalysts and improving the circularity of both waste streams.

## Figures and Tables

**Table 1:** Comparison of different methods for plastic waste recycling.

Method	Feedstock Tolerance	Product Quality	Circularity Potential	Economic Maturity	Main Advantage	Main Limitation	References
Mechanical recycling	Low–medium	Medium	Medium–high	High	Low-cost and mature	Requires clean sorted plastics	[12]
Solvent-based purification	Medium–high	High	High	Medium	Removes additives/contaminants	Solvent recovery and cost	[13]
Depolymerization/solvolysis	Low–medium	Very high	Very high	Medium	Monomer recovery	Polymer-specific chemistry	[14]
Pyrolysis	High	Medium	Medium	Medium–high	Treats mixed plastics	Energy demand and oil upgrading	[15]
Gasification	Very high	Medium	Medium	Medium	Converts waste to syngas	High temperature and gas cleaning	[16]
Biological/enzymatic recycling	Low	Potentially high	Potentially high	Low–medium	Mild and selective	Slow and polymer-specific	[17]

**Table 2:** Proximate and ultimate analysis of major plastics.

	Moisture, %	VM, %	Ash, %	FC, %	C, %	H, %	N, %	S, %	Cl, %	O, %	Reference
HDPE	0.215	98.35	0.212	1.223	82.86	16.85	0.00	0.28	0.00	0.01	[30]
HDPE	0.1 ± 0.1	77.7 ± 1.6	10.4 ± 0.2	11.9 ± 1.6	73.33	13.28	0	NR	NR	0	[31]
LDPE	0.189	98.65	0.149	1.012	83.32	16.42	0.00	0.24	0.00	0.02	[30]
LDPE	0.1 ± 0.1	99.8 ± 0.6	0.0 ± 0.0	0.2 ± 0.6	83.67	16.33	0	NR	NR	0	[31]
PVC	0.299	88.66	7.571	3.470	37.78	4.83	0.14	0.16	56.73	0.36	[32]
PET	0.007	88.99	2.092	8.911	87.98	10.45	0.60	0.14	0.00	0.83	[30]
PET	0.4 ± 0.3	85.7 ± 1.9	8.3 ± 0.1	6.0 ± 1.9	62.95	4.12	0	NR	NR	32.93	[31]
PP	0.069	99.57	0.280	0.081	84.92	14.75	0.04	0.23	0.00	0.06	[30]
PP	0.1 ± 0.1	97.7 ± 0.1	0.0 ± 0.0	2.3 ± 0.1	83.25	16.75	0	NR	NR	0	[31]
PS	0.201	98.18	0.168	1.451	88.51	11.42	0.00	0.02	0.00	0.05	[30]
PS	1.0 ± 0.4	98.8 ± 0.3	0.0 ± 0.0	1.2 ± 0.3	89.81	7.48	2.71	NR	NR	0	[31]

**Table 6:** Types of processes for SS processing [70].

Type	Products Yield, wt%			References
	Gases	Liquid	Solid	
Pyrolysis	2–29	5–20	10–80	[71,72,73]
Gasification	56–63	0–2	30–40	[74]
Hydrothermal carbonization (HTC)	1–3	26–30	64–85	[75,76,77,78]
Hydrothermal liquification (HTL)	6–13	31–48	4.7–40	[79,80,81]

**Table 8:** SS char BET characteristics [138,139].

Article	Temperature, °C	BET Surface Area, m^2^·g^−1^	Pore Volume cm^3^ · g^−1^
Kanafin et al.	300	3.649	n/d
	500	87.453	0.074
	700	92.33	0.079
	900	83.801	0.101
Sierra et al.	600	58.4	0.144
	700	50.7	0.143
	800	113	0.156
	900	43.1	0.141
	1000	3.5	0.003

**Table 9:** Chemical analysis of biochar, wt% [143,144,145,146].

Article	Na_2_O	MgO	Al_2_O_3_	SiO_2_	P_2_O_5_	K_2_O	CaO	TiO_2_	Fe_2_O_3_
Kritikaki et al., 2025 [143]	11.9	5.6	3.3	10.5	17.5	0.8	17.8	0.8	10.1
Mohammadi et al., 2024 [144]	—	3.39	5.23	25.67	13.43	1.78	17.45	—	6.38
Babayiğit et al., 2024 [145]	0.36	2.24	11.93	34.89	8.62	3.07	6.57	0.93	4.64
Qu et al., 2023 [146]	—	1.25	2.19	7.37	7.93	—	1.14	2.02	63.02

**Table 10:** Effect of Sewage Sludge-Plastic Co-Processing on gas and Tar Characteristics.

System	Main Effect on Gas Production	Effect on Tar/Condensables	Effect on Tar Composition	Ref.
SS + PE, PP or PS, 600 °C pyrolysis followed by steam reforming at 700–900 °C	Plastics increased H_2_, CH_4_ and light hydrocarbons, PE gave strongest H_2_ enhancement	Higher reforming temperature strongly decreased tar, 50% PP or PS gave optimal gasification efficiency at 800 °C	SS-derived oxygenates were cracked; at high plastic fractions, C_12_–C_20_ hydrocarbons and C_21+_ waxes could resist conversion	[86]
SS pyrolysis at 450 °C coupled to PP conversion at 600–800 °C	Highest gas formation at 450/800 °C	Condensable yield decreased strongly at 800 °C	Aromaticity decreased at 700 °C but increased at 800 °C, including heavier multi-ring aromatics	[171]
SS + simulated MSW containing PP and PE, two-stage fluidized bed with AC, Ni/AC or Fe/AC	Ni/AC increased H_2_ to 31.89 mol%	Steam and metal-loaded AC promoted tar reforming	Not quantitatively characterized	[89]
SS and plastic labels evaluated separately for potential blending	Plastic labels gave high-energy, CH_4_-rich gas	No direct co-feed tar result	Not analyzed for an actual SS–plastic blend	[172]

## Data Availability

No new experimental data were generated in this study. The data discussed in this review were obtained from the cited publications and publicly available sources. The compiled information is presented in the article and its appendices, with the original sources identified through the corresponding references.

## Abbreviations

The following abbreviations are used in this manuscript:

ABS	Acrylonitrile butadiene styrene
AC	Activated carbon
BET	Brunauer–Emmett–Teller
BTEX	Benzene, toluene, ethylbenzene, and xylenes
DEHP	Di(2-ethylhexyl) phthalate
DSS	Digested sewage sludge
EPS	Extracellular polymeric substances
FCC	Fluid catalytic cracking
FTIR	Fourier-transform infrared spectroscopy
FWO	Flynn–Wall–Ozawa
GC–MS	Gas chromatography–mass spectrometry
HDPE	High-density polyethylene
HHV	Higher heating value
HIPS	High-impact polystyrene
HTC	Hydrothermal carbonization
HTL	Hydrothermal liquefaction
KAS	Kissinger–Akahira–Sunose
LDPE	Low-density polyethylene
LHV	Lower heating value
MSC	Municipal sludge char
MSW	Municipal solid waste
OECD	Organisation for Economic Co-operation and Development
PA6	Polyamide 6
PAHs	Polycyclic aromatic hydrocarbons
PC	Polycarbonate
PCB/PCBs	Polychlorinated biphenyl/polychlorinated biphenyls
PCDD/F/PCDD/Fs	Polychlorinated dibenzo-*p*-dioxins and dibenzofurans
PE	Polyethylene
PET	Polyethylene terephthalate
PFAS	Per- and polyfluoroalkyl substances
PP	Polypropylene
PS	Polystyrene
PUR	Polyurethane
PVC	Polyvinyl chloride
PyChar	Pyrolysis char
PyGas	Pyrolysis gas
PyOil	Pyrolysis oil
RDF	Refuse-derived fuel
SDS	Sodium dodecyl sulfate
SS	Sewage sludge
SSA	Sewage sludge ash
SSC	Sewage sludge char
TA	Terephthalic acid
TCLP	Toxicity Characteristic Leaching Procedure
TEA	Techno-economic analysis
TGA	Thermogravimetric analysis
UDSS	Undigested sewage sludge
VOC/VOCs	Volatile organic compound/volatile organic compounds
WtE	Waste-to-energy
XPS	X-ray photoelectron spectroscopy
XRD	X-ray diffraction
XRF	X-ray fluorescence
HZSM-5	Proton-form ZSM-5 zeolite
ZSM-5	Zeolite Socony Mobil-5
HUSY	Proton-form ultrastable Y zeolite
HY/H-Y	Proton-form Y zeolite
Hβ/H-Beta	Proton-form Beta zeolite
HMOR	Proton-form mordenite
MCM-41	Mobil Composition of Matter No. 41
SBA-15	Santa Barbara Amorphous No. 15
SAPO-11	Silicoaluminophosphate-11
Al-MCM-41	Aluminum-containing MCM-41
Al-SBA-15	Aluminum-containing SBA-15
Ni/AC	Nickel supported on activated carbon
Fe/AC	Iron supported on activated carbon
Fe/MSC	Iron supported on municipal sludge char
Ni/MSC	Nickel supported on municipal sludge char
Fe–Ni/MSC	Iron–nickel supported on municipal sludge char
Ni/SSC	Nickel supported on sewage sludge char
Re–Ni/SSC	Rhenium–nickel supported on sewage sludge char
Ni/ZSM-5	Nickel-supported ZSM-5
Ni/SAPO-11	Nickel-supported SAPO-11
CoMo/Al_2_O_3_	Cobalt–molybdenum supported on alumina
NiMo/Al_2_O_3_	Nickel–molybdenum supported on alumina
A	Ash
FC	Fixed carbon
VM	Volatile matter
W	Moisture/water content
db	Dry basis
NR	Not reported
n/d	Not determined/no data reported
ISSA	Incinerated sewage sludge ash

## Appendix A

**Table A1:** SS utilization routes.

Method	Main Purpose	Feedstock	Main Products	Advantages	Limitations	References
Landfilling	Final disposal of sludge	Dewatered/stabilized SS	Landfilled waste, landfill gas	Simple, widely available, low technical complexity	Land occupation, CH_4_ emissions, leachate, loss of resources, increasingly restricted	[65,69,211]
Agricultural use	Nutrient and organic-matter recycling	Stabilized biosolids/treated SS	Soil amendment, fertilizer-like material	Returns N, P, organic matter and micronutrients to soil	Risk of heavy metals, pathogens, PFAS, pharmaceuticals, microplastics, strict regulation required	[38,67,212,213,214,215]
Composting	Biological stabilization and humification	Dewatered SS, often mixed with bulking agents	Compost-like product	Reduces odor/pathogens, improves handling, produces soil amendment	Long processing time, land demand, possible metal/organic contaminant accumulation	[68,214]
Anaerobic digestion	Organic-matter stabilization and energy recovery	Primary/secondary sludge, thickened SS	Biogas, digestate	Produces renewable energy, reduces volatile solids, mature technology	Digestate still requires management, incomplete degradation of recalcitrant organics, sensitivity to operating conditions	[216,217]
Incineration	Volume reduction, pathogen/toxin destruction, ash production	Dried or mechanically dewatered SS	SS ash, heat, flue gas	Strong volume reduction, destroys pathogens and organic pollutants, ash can be used for P recovery	High energy demand for drying, air emissions, ash/heavy-metal management	[18,40,59,66,218]
Pyrolysis	Thermochemical conversion into carbonaceous products	Dried/dewatered SS	Biochar/char, bio-oil, gas	Produces char usable as adsorbent/catalyst precursor, lower O_2_ demand than incineration, potential pollutant immobilization	Requires drying, variable product quality, tar/oil treatment, heavy metals remain in solid phase	[34,71]
Gasification	Conversion of organic fraction into fuel gas	Dried/dewatered SS	Syngas, ash/slag, tar	Produces combustible gas, high energy recovery potential, lower solid residue than raw sludge	Tar formation, gas cleaning, ash melting/agglomeration, feedstock moisture sensitivity	[18,58]
Hydrothermal carbonization (HTC)	Conversion of wet sludge into carbon-rich solid	Wet SS/high-moisture sludge	Hydrochar, process water, gas	Suitable for wet feedstock, avoids full drying, produces hydrochar	Process water treatment required, hydrochar quality varies, technology less mature than incineration/AD	[18]
Hydrothermal liquefaction (HTL)	Production of liquid fuel-like fraction from wet sludge	Wet SS/high-moisture sludge	Biocrude, aqueous phase, gas, solid residue	Can process wet sludge, produces energy-dense biocrude	Complex product upgrading, aqueous phase toxicity, high pressure, lower maturity	[18,44,70]
Phosphorus recovery	Recovery of nutrients, mainly P	SS, digestate, SS ash, liquid streams	Phosphate salts, struvite, recovered P products	Supports circular nutrient economy, reduces dependence on phosphate rock	Requires chemical extraction/precipitation, heavy-metal separation, cost and purity issues	[19,57,58,59]
Construction material production	Material recovery and replacement of mineral raw materials	SS ash, incinerated SS ash	Cement additive, concrete, bricks, ceramics, aggregates	Reduces landfill demand and raw material use, useful route for ash valorization	Leaching risk, variable SSA composition, possible strength/water-demand issues	[69]

**Table A2:** Comparative assessment of mono-conversion and co-conversion of plastic waste and sewage sludge.

Technology	Plastic-Only Conversion	SS-Only Conversion	Potential Benefit of SS–Plastic Co-Conversion	Main Limitation	References
Incineration	Polyolefins have a high calorific value, whereas aromatic polymers require higher energy input for combustion	Low heating value and high moisture content	Reduced formation of persistent organic pollutants; increased calorific value of the feedstock mixture; improved flowability and processability	Increased pollutant emissions (e.g., NO_x_, SO_2_, CO, HCl, and HF); need for pretreatment and dewatering; heavy-metal emissions; high ash yield	[93,101,111,219]
Pyrolysis	Hydrocarbon-rich liquids or gases, depending on polymer	Oxygenated bio-oil, gas and mineral-rich char	Potential improvement in pyrolysis-oil quality; high yield of the gasoline-range fraction; stabilization of the biochar carbon framework; increased energy recovery efficiency of up to 64%	Need for pretreatment; product dilution; ash accumulation	[84,94,128,220,221]
Gasification	Production of combustible gas and syngas	Syngas, inorganic residues, and tar	Feedstock complementarity and potential non-additive enhancement of H_2_ formation and tar conversion	Heavy hydrocarbon formation; ash-related issues; need for gas cleaning to remove nitrogen- and sulfur-containing compounds and other toxic contaminants	[87,222,223]
Hydrothermal carbonization	Polymer-dependent hydrothermal transformation, hydrochar and CO_2_ formation	Hydrochar production from wet sludge	Co-treatment of wet SS with reactive plastics and modification of hydrochar characteristics	Limited conversion of PE, PP and PS under conventional HTC conditions	[77,181,185]
Hydrothermal liquefaction	Polymer-dependent liquid production or depolymerization	Biocrude production from wet SS	Integrated processing, modification of biocrude composition	Limited direct evidence, phase separation and heteroatom contamination	[190,224,225,226]

**Table A3:** Previous studies on PE, PP, PET, and PS pyrolysis.

Polymer and Comparison	Reactor Type	Catalyst Use	Temperature	Heating Rate and Residence Time	Reported Product Distribution	Effect of Temperature/Heating Rate	Reference
PP, non-catalytic, representative cross-study points	Batch reactor at 380 °C, micropyrolyser at 600 °C, fluidized bed at 760 °C	None	380, 600 and 760 °C	3 °C min^−1^ at 380 °C, heating rate and 5 s residence at 600 °C, at 760 °C	380 °C: oil 80.1%, gas 6.6%, char 13.3%, 600 °C: oil 68.0%, gas 30.36%, char 1.64%, 760 °C: oil 42.2%, gas 55.8%, char 1.8%	The cross-study results show the expected shift from liquid/solid products toward gas as thermal severity increases. However, the simultaneous change in reactor type means this should not be interpreted as a controlled temperature series.	[115,227,228,229]
HDPE, non-catalytic temperature increase	Reactor type NR in the review summary	None	400 → 450 °C	NR	Oil increased from 22.5 to 40.5%	Raising temperature initially enhanced chain scission and conversion of HDPE into condensable products. The tested range apparently remained below the temperature at which secondary vapor cracking became dominant.	[230]
HDPE, non-catalytic high-temperature series	Reactor type NR in the review summary	None	500 → 800 °C	NR	Oil decreased from 48 to 19%, gas production increased	Above the liquid-yield optimum, secondary cracking converted condensable hydrocarbons and waxes into lighter non-condensable gases.	[231]
LDPE, slow pyrolysis	Fixed-bed reactor	None	500 °C	6 °C min^−1^, residence time NR	Oil 80.41%, gas 19.43%, char 0.16%	A moderate temperature and slow heating rate produced a predominantly liquid product with negligible solid residue.	[232]
LDPE, fast pyrolysis	Infrared-heated fixed-bed reactor	None	550 °C	20 °C s^−1^, residence time NR	Oil 93.42%, gas 6.36%, char 0.22%	Rapid heating can still produce very high liquid yield when heat transfer is efficient and primary vapors are rapidly removed and condensed. Thus, heating rate alone does not determine whether oil or gas dominates.	[233]
LDPE/HDPE/PP mixture, controlled heating-rate series	Reactor type NR in the review summary	None	410 °C	10 °C min^−1^ and 60 min, 19 °C min^−1^ and 43 min, 28 °C min^−1^ and 25 min	Oil: 69, 59 and 43%, respectively	At 410 °C, reducing the heating rate while increasing feedstock residence time increased liquid recovery. Slower heating allowed more complete conversion without imposing severe vapor cracking.	[234]
LDPE/HDPE/PP mixture at higher temperature	Reactor type NR in the review summary	None	550 °C	10–28 °C min^−1^, residence time decreased from 60 to 25 min	Gas increased from 40% at 10 °C min^−1^ to 75% at 28 °C min^−1^	Increasing both temperature and heating rate shifted the distribution strongly toward gas. At 550 °C, secondary cracking was much more important than at 410 °C.	[234]
PP, catalytic temperature series	Fixed-bed reactor	ZSM-5	500, 520, 560, 580 and 640 °C	NR	Oil: 20, 43, 68, 65 and 40%, respectively, gas increased from 35% at 580 °C to 65% at 640 °C	Oil yield reached a maximum at approximately 560 °C. Further heating intensified catalytic and thermal cracking of vapors into permanent gases.	[235]
HDPE, catalyst comparison at constant temperature	Continuous horizontal-tube reactor, feed rate 9 kg h^−1^	None versus 5% ZSM-5	520 °C	NR	No catalyst: gasoline 18.2%, light oil 17.9%, ZSM-5: gasoline 34.5%, light oil 24.1%	At the same temperature and reactor conditions, ZSM-5 increased the reported gasoline and light-oil fractions, indicating additional cracking and upgrading of heavy HDPE vapors.	[236]
PP, catalyst comparison at constant temperature	Continuous horizontal-tube reactor, feed rate 9 kg h^−1^	None versus 5% ZSM-5	520 °C	NR	No catalyst: gasoline 33.5%, light oil 22.5%, ZSM-5: gasoline 53%, light oil 30%	ZSM-5 considerably increased the lighter gasoline and oil fractions. The effect was attributable to catalytic cracking and isomerization rather than heating rate.	[236]
HDPE, two-stage catalytic pyrolysis	Two-stage fixed-bed reactor	ZSM-5/MCM-41, 1:1, feedstock-to-catalyst ratio 1:2	500 °C	10 °C min^−1^	Oil 83.15%, aromatics 95.85% of oil, gasoline-range compounds 97.72% of aromatics	Moderate heating combined with sequential mesoporous and acidic catalytic cracking gave a high oil yield and exceptionally high aromatic/gasoline-range selectivity.	[237,238]
PE/PP mixture, catalytic pyrolysis	Fixed-bed reactor	HZSM-5	450 °C	NR	Oil 23.8%, gas 75.1%, char 1.1%	Even at a moderate temperature, strongly acidic HZSM-5 shifted most of the converted material toward gas, demonstrating that catalyst acidity and pore structure can outweigh the nominal temperature effect.	[239]
PS	Laboratory electrically heated batch reactors	None	418–500 °C	1–6 °C min^−1^, 60–240 min	Oil: 78.0–80.7%	Across conventional batch studies, moderate temperatures and slow heating produced high liquid yields. PS depolymerization favors styrene-rich, non-waxy aromatic oil. Because several studies are pooled, the narrow oil-yield range cannot be attributed solely to temperature.	[240,241,242]
PS	Microwave-heated laboratory batch reactors	No conventional catalyst, microwave absorbers were used in some studies	330–535 °C	Approximately 49 °C min^−1^, 5.5–60 min	Oil: 93.0–98.8%	Rapid internal microwave heating and relatively short processing times produced exceptionally high liquid recovery. Efficient heating and rapid vapor removal limited secondary conversion to permanent gases.	[241,243,244]
PS	Parr benchtop autoclave	None	500 °C	5 °C min^−1^, 60 min	Liquid: 71%, gas: 2%, residue: 27%	Slow heating at 500 °C produced predominantly liquid oil, although the comparatively high residue indicates that autoclave hydrodynamics, long solid residence, and feed characteristics also influenced the balance.	[245]
PS	Pilot-scale batch reactor	None	450 °C	10 °C min^−1^, 75 min	Liquid: 81%, gas: 13%, char: 6%	At 450 °C, PS was efficiently converted to liquid products. Compared with the 500 °C autoclave result, the higher liquid yield may reflect both lower thermal severity and improved collection rather than temperature alone.	[246]
PS	Fluidized-bed reactor	None	515 °C	Heating rate NR, vapor residence time ≈0.025 s	Oil: 99.7%	Very short vapor residence sharply suppressed secondary cracking, gas formation, and aromatic condensation. This example shows that high temperature can still maximize liquid yield when vapors are removed and quenched almost immediately.	[247]
PS	Laboratory batch reactor	Zn, approximately 17wt%, in situ	450 °C	Heating rate NR, 120 min	Oil: 97%	Zinc promoted PS depolymerization and conversion to condensable aromatic products at a moderate temperature. The very high oil yield reflects catalytic action and therefore should not be compared directly with non-catalytic temperature series.	[240]
PS	Laboratory electrically heated batch/two-stage reactor	MgO, 5wt%	Pyrolysis: 500 °C, catalytic bed: 500 °C in ex situ mode	10 °C min^−1^, residence time NR	In situ: oil 78%, ex situ: oil 89%	At the same nominal temperature and heating rate, ex situ vapor upgrading gave more liquid than direct catalyst–plastic contact. Preheating the downstream catalyst reduced crosslinking and promoted cracking of vapors into condensable products.	[241,248]
PS	Pilot batch reactor	Thermally modified natural zeolite, approximately 9wt%, in situ	450 °C	10 °C min^−1^, 75 min	Oil: 70%	The catalyst reduced liquid yield relative to non-catalytic PS at similar temperature because part of the styrene-rich vapor underwent additional cracking and gas formation. The remaining oil was generally more aromatic and lighter.	[249]
PS	Catalytic reforming system, reactor geometry not specified in the review	HY zeolite	400 → 450 °C	NR	At 450 °C, liquid ≈80%, benzene and toluene increased, while styrene, styrene dimer, ethylbenzene, and PAHs decreased	Increasing reforming temperature enhanced cracking of heavier aromatic compounds and shifted PS oil away from styrene and oligomers toward lighter monocyclic aromatics.	[250]
PET	Laboratory electrically heated batch reactor slow pyrolysis	None	450–600 °C	5 °C min^−1^, vapor residence ≈0.33 s or feed residence up to 60 min	Waxy oil: 56–67%	PET produced a waxy, oxygenated condensate rather than a conventional hydrocarbon oil. Increasing temperature promotes ester-bond cleavage, decarboxylation, and decarbonylation, but also increases CO, CO_2_, and secondary aromatic formation.	[251,252]
PET	Parr benchtop autoclave slow pyrolysis	None	500 °C	5 °C min^−1^, 60 min	Liquid: 15%, gas: 32%, residue: 53%	At moderate heating and prolonged residence, PET gave little recoverable liquid and considerable residue. Its oxygen-rich structure favored CO/CO_2_ release, acidic products, and carbonization reactions.	[245]
PET	Conical spouted-bed reactor with sand fast pyrolysis	None	500 °C	Rapid particle heating, PET feed 1.5 g min^−1^, vapor residence NR	Liquid: 14%, gas: 44%, solid/residue: 42%	Improved heat transfer did not substantially increase PET liquid yield. Rapid devolatilization instead increased gas production, while terephthalic- and benzoic-acid-derived species contributed to solids and deposits.	[253]
PET	Laboratory batch reactor with downstream ex situ catalyst	Sulfated zirconia, approximately 3wt%	525 °C	45 °C min^−1^, vapor residence ≈0.33 s	Waxy oil: 52%	The high heating rate rapidly generated vapors, while sulfated zirconia promoted cracking and benzoic-acid recovery. The lower condensate yield than the NiCl_2_ case reflects stronger secondary conversion and different catalyst operation.	[254]

**Table A4:** List of catalysts reported for the catalytic pyrolysis of PE, PP, PS, PET, and PVC.

Catalyst/Catalytic Material	Catalyst Class	Plastic(s) Studied	Main Reported Role/Effect	Reference(s)
HZSM-5 (ZSM-5)	Microporous zeolite	PE, PP, PS, PET, mixed plastics	Strong cracking and aromatization; favors lighter hydrocarbons, gases and aromatics. Excessive acidity can promote secondary cracking and coke.	[123,255]
HUSY zeolite	Faujasite-type zeolite	PE, HDPE, mixed PE/PP/PS/PET	Promotes cracking of heavy fractions and suppresses wax formation; can favor monoaromatics.	[123,256,257]
H-Y zeolite	Faujasite-type zeolite	PE, PP, PS	Converts polymers into lighter hydrocarbons; its large pores facilitate access of relatively bulky intermediates.	[258,259]
Hβ (H-Beta)	Large-pore zeolite	PE, HDPE	Promotes cracking while providing greater accessibility for larger pyrolysis intermediates than narrow-pore zeolites.	[255,258]
Mordenite (HMOR)	Zeolite	PE, HDPE, PS	Promotes cracking and aromatization, although its one-dimensional pore structure may impose diffusion limitations.	[123,258,260]
FCC catalyst	Zeolite-containing commercial catalyst	PE, PP, PS, mixed plastics	Strong cracking of heavy hydrocarbons and waxes toward lighter fuel-range products and gases.	[123,261,262]
Spent FCC catalyst	Waste-derived FCC catalyst	PE, PP, PS	Low-cost catalyst capable of high conversion and liquid yields; activity depends on residual acidity and previous catalyst history.	[263,264]
Silica–alumina (SiO_2_–Al_2_O_3_)	Amorphous solid acid	PE, PP, mixed plastics	Brønsted and Lewis acidity promotes cracking; generally less shape-selective than zeolites.	[123,265]
MCM-41/Al-MCM-41	Mesoporous (alumino)silicate	PE, PP	Large pores improve accessibility of bulky intermediates; Al incorporation increases acidity and cracking activity.	[122,266]
Al-SBA-15	Mesoporous aluminosilicate	PP	Promotes degradation at lower temperature and conversion toward lighter hydrocarbons and gases.	[122]
Metal/SBA-15 (Al, Co, Ni, Zn)	Metal-modified mesoporous silica	HDPE/polyolefins	Improves accessibility and allows product selectivity to be tuned through the supported metal.	[123]
Zn/SBA-15	Zn-modified mesoporous catalyst	HDPE/polyolefins	Particularly effective for producing shorter-chain hydrocarbons with relatively low aromatic selectivity.	[123]
MgO	Basic metal oxide	Plastic wastes/polyolefins	Low-cost catalyst that can reduce degradation temperature and modify oil/gas distribution.	[122,267]
Zn, ZnO, ZnCl_2_	Metal/metal compounds	PS	Strongly influences PS depolymerization and product selectivity; Zn gave particularly high liquid yield in one reported study.	[125,268]
Kaolin	Natural clay/aluminosilicate	HDPE, PS	Low-cost, moderately acidic catalyst; promotes cracking while generally avoiding the severe over-cracking associated with strongly acidic zeolites.	[269,270]
Bentonite	Smectite clay	HDPE, PP, PS	Promotes liquid formation and cracking; catalytic properties can be substantially enhanced by acid treatment or metal modification.	[271,272,273]
Ni/acid-washed bentonite	Metal-modified clay	PP	High catalytic activity and high liquid yield reported for PP pyrolysis.	[271,274]
Pillared/modified natural clays	Modified clay catalysts	PE, HDPE, mixed plastics	Larger accessible pores and enhanced acidity improve cracking of bulky polymer-derived molecules.	[275,276]
Bentonite clay (Gachi clay)	Natural clay	LDPE	Low-cost catalyst for conversion of LDPE into fuel-range products.	[277]
Pyrophyllite/halloysite	Natural clay minerals	PS	Promote PS conversion, with acid-treated pyrophyllite showing high aromatic and styrene selectivity.	[278]
CaO	Basic metal oxide	PS	Reported as an inexpensive catalyst capable of promoting PS depolymerization and styrene-rich products.	[123,125]
Sulphated zirconia	Strong solid-acid catalyst	PET	Promotes selective conversion of PET pyrolysis products, particularly recovery of benzoic acid.	[254]
Modified natural zeolite	Natural zeolite	PE, PP, PS, PET	Thermal and acid activation improves catalytic activity, and promotes aromatic-rich pyrolysis oils from different plastics.	[249]
Ni/ZSM-5	Metal-modified zeolite	Chlorine-contaminated/PVC-containing plastic waste	Catalytic vapor upgrading and modification of pyrolysis-oil composition in chlorine-containing feeds.	[279]
Ni/SAPO-11	Metal-modified silicoaluminophosphate	Chlorine-contaminated/PVC-containing plastic waste	Used for in situ catalytic upgrading of vapors from chlorine-containing plastic waste.	[279]
Red mud	Waste-derived mineral catalyst	Mixed plastics, PVC-containing waste	Low-cost cracking catalyst; its alkaline/mineral components may additionally contribute to contaminant management.	[279,280]
Ca(OH)_2_	Basic sorbent/additive	PVC-containing plastic waste	Primarily functions as an HCl/chlorine scavenger rather than a conventional cracking catalyst.	[279]

**Table A5:** Experimental evidence of the effects of sewage sludge incorporation on thermochemical plastic conversion.

Study	Technology	Feedstock	Operating Conditions	Reference Case	SS–Plastic Mixture	Observed Effect
(1) Jadlovec et al. [93]	Co-incineration	SS + predominantly PE automotive waste	Pelletized fuels; fluidized-bed combustion; stable operation at ca. 915–939 °C	Pure SS and pure PE were experimentally combusted under the same study framework	SS/PE = 1:1	The blend had an LHV of 16.29 MJ kg^−1^, compared with 9.83 MJ kg^−1^ for SS and 31.81 MJ kg^−1^ for PE. CO decreased to 192 mg vs. 612 mg for SS and 1213 mg for PE. Relative to SS, the blend strongly reduced HCl (153.5 vs. 3226 mg) and SO_2_ (24,319 vs. 58,840 mg), but NO_x_ remained high (13,331 vs. 13,865 mg for SS and 1477 mg for PE). Thus, plastic addition clearly improves the energetic properties of SS, but the blend does not outperform pure PE energetically and retains sludge-related emission constraints.
(2) Gandon-Ros et al. [111]	Co-incineration	PVC-containing e-waste + SS	Laboratory tubular reactor; 850 °C; O_2_ ratio λ = 0.15 or 0.50; inhibitor-to-Cl molar ratio R_i_ = 0.25–0.75	PVC e-waste combustion without the corresponding SS-derived N/S inhibitor contribution	PVC e-waste + SS at controlled N+S/Cl ratios	This is one of the clearest cases where SS provides a benefit to plastic conversion itself. Under oxygen-rich conditions (λ = 0.50), SS suppressed chlorinated persistent organic pollutants; at R_i_ = 0.75, PCDD/F formation was reduced by almost 100% and dl-PCBs by approximately 95%. The benefit was attributed to N- and S-containing sludge species acting as in situ inhibitors.
(3) Zhu et al. [108]	Co-incineration/integrated thermal treatment	Pre-dried SS + waste plastic	Pilot screw-type two-chamber incinerator; sequential drying, pyrolysis, gasification and combustion	Performance limitations of sludge combustion and plastic combustion considered separately; no rigorous theoretical additive baseline was reported	Pre-dried SS–plastic blend	Plastic addition enabled self-sustaining incineration of the sludge–plastic mixture, with the secondary combustion chamber reaching approximately 1000 °C and complete combustion. This study therefore provides strong process-feasibility evidence, but weaker evidence of true chemical synergy because a quantitative additive control was not established.
(4) Zaker et al. [21]	Co-pyrolysis	SS + LDPE	TGA at multiple heating rates; principal blend decomposition ca. 200–600 °C; several SS:LDPE ratios	Experimental pure SS and pure LDPE; theoretical TGA/DTG curves calculated by weighted addition	Particularly SS/LDPE = 1:1 and 1:2	Experimental TGA/DTG curves deviated from theoretically additive behavior, demonstrating chemical interaction. The 1:1 blend was identified as the most favorable because of a higher pyrolysis index and a shift in T_max_ toward lower temperature. Activation-energy analysis also indicated favorable interaction. This is genuine non-additive evidence rather than simply comparison with SS alone.
(5) Ling and Li [129]	Co-pyrolysis	SS + PP; SS + HDPE	TGA/TG-MS; polyolefin fractions 20, 50 and 80 wt%; heating rates 10–50 °C min^−1^	Pure SS, pure PP and pure HDPE together with calculated/model-based kinetic behavior	SS/PP and SS/HDPE at 20–80 wt% plastic	Co-pyrolysis produced higher volatile conversion and degradation rates and lower activation energies than expected from the individual feedstocks. Evolved-gas analysis supported enhanced dehydration and decarboxylation caused by interactions between polyolefin-derived H radicals and oxygenated SS intermediates. The study therefore provides strong kinetic evidence for genuine synergistic interaction.
(6) Qi et al. [131]	Co-pyrolysis	PET + SS	PET/SS co-pyrolysis with kinetic, char and EPFR characterization	PET-only pyrolysis/char and co-pyrolysis response	PET/SS = 1:1 was a key evaluated mixture	At 1:1, EPFR concentration in PET-derived char was reduced by 38.86–48.55%. The reported activation energy decreased by 78.83%, while biochar yield rate increased by 30.93% because of synergistic effects. At 400 °C, SS addition increased the specific surface area of PET-based char by 43.62-fold and promoted aromatization/defect development. This is unusually strong evidence that SS can materially alter PET conversion rather than acting solely as an inert diluent.
(7) Yang et al. [86]	Co-gasification	SS + PE; SS + PP; SS + PS	TGA plus two-stage fixed bed; primary conversion at ca. 600 °C, secondary steam-gasification/reforming at 700–900 °C; plastic fractions 25, 50 and 75 wt%	Pure SS and pure PE/PP/PS plus theoretically additive interaction analysis	SS 75%/plastic 25%, SS 50%/plastic 50% and SS 25%/plastic 75%	The interaction was strongly dependent on temperature, polymer and ratio. Co-gasification produced positive deviations in H_2_ and CH_4_ and lower-than-additive C_n_H_m_, supporting enhanced secondary cracking. PE provided the strongest H_2_ enhancement. At 800 °C, the best gasification efficiency among PP and PS blends occurred at about 50 wt% plastic. Crucially, the study shows that synergy is not universally positive: some temperature/ratio regions showed inhibition.
(8) Lu et al. [184]	Co-HTC	SS + PVC	230 °C, 1 h; PVC proportion approximately 30–70 wt%	HTC of individual PVC and SS/expected individual behavior	SS/PVC mixtures; SS/PVC = 7:3 highlighted	Compared with HTC of PVC alone, the presence of SS increased PVC dechlorination to approximately 69.49–81.35%. At SS/PVC = 7:3, hydrochar reached an HHV of 13.58 MJ kg^−1^ with favorable combustion behavior. The interaction also affected Zn, Mn, Ni and As migration because PVC-derived HCl acidified the aqueous phase. Thus, sludge can improve dechlorination, but metal mobility becomes an important trade-off.
(9) Xie et al. [185]	Co-HTC	SS + PVC	Optimization around 240 °C, 1.5 h; water loading up to 0.84 g cm^−3^; varying PVC proportion	SS/PVC systems at different plastic and water loadings; authors explicitly evaluated synergistic behavior	Optimized SS/PVC co-HTC system	Appropriate PVC loading produced a positive synergistic effect on denitrification efficiency and hydrochar yield. Under optimized conditions, hydrochar contained only 0.94 wt% N with an HHV of 9.84 MJ kg-1. Increasing water loading from 0.54 to 0.84 g cm^−3^ decreased hydrochar N from 1.50 to 0.94 wt% and increased denitrification efficiency from 60.11 to 75.00%. Excessive PVC, however, promoted graphite-like carbon and could hinder further combustion improvement.
(10) Prestigiacomo et al. [198]	HTL—indirect evidence from real SS containing microplastics	Real municipal SS containing ca. 3 wt% microplastics	325 °C, 30 min; uncatalyzed and CoMo/Al_2_O_3_, NiMo/Al_2_O_3_ or activated-carbon-felt catalysis	Uncatalyzed real sludge and controlled hydrothermal experiments with model PVC, PE and nylon	Microplastics inherently present in SS rather than deliberately prepared bulk SS/plastic mixtures	Catalytic HTL generated a separate white wax phase corresponding to 5, 8 and 6 wt% daf with CoMo/Al_2_O_3_, NiMo/Al_2_O_3_ and ACF, respectively. The phase contained oleamide and chlorinated organic compounds, negatively affecting biocrude quality. This should not be presented as evidence of beneficial SS–plastic synergy; instead, it demonstrates that plastics materially alter SS-HTL phase behavior and highlights the lack of controlled bulk-plastic/SS co-HTL studies.
